# Delayed late ventricular depolarization is associated with inflammation in epicardial Fat Among females: a New avenue in cardiac electrophysiology

**DOI:** 10.3389/fcvm.2026.1947506

**Published:** 2026-09-07

**Authors:** Nadeem A. Ahmed, David Sánchez-López, Rumeysa Basdas, José Ramón Núñéz-Caamaño, José Manuel Martínez-Cereijo, Laura Reija-López, Ángel L. Fernández, Javier García-Seara, Moisés Rodriguez-Mañero, José R. González-Juanatey, Sonia Eiras

**Affiliations:** 1 Translational Cardiology, Health Research Institute of Santiago de Compostela (IDIS), Santiago de Compostela, Spain; 2 University of Santiago de Compostela (USC), Santiago de Compostela, Spain; 3 Cardiovascular Area, Centre for Research in Molecular Medicine and Chronic Diseases (CIMUS), University of Santiago de Compostela, Santiago de Compostela, Spain; 4 Biomedical Research Network, Cardiovascular Disease, CIBERCV, ISCIII, Madrid, Spain; 5 Cardiac Surgery Department, Complexo Hospitalario Universitario de Santiago de Compostela, Santiago de Compostela, Spain; 6 Cardiology Department, Complexo Hospitalario Universitario de Santiago de Compostela, Santiago de Compostela, Spain

**Keywords:** epicardial adipose tissue, inflammation, late ventricular depolarization, S-wave upstroke time, terminal activation duration

## Abstract

**Background:**

Epicardial adipose tissue (EAT) has been linked to cardiac conduction through direct myocardium interaction and endocrine activity. We investigated sex-specific associations between ventricular electrical activity and the molecular composition of EAT.

**Methods:**

EAT biopsies from 146 cardiac surgery patients were analysed for inflammatory cell markers (CD16, DEFA3, CXCR2, CD68, CD14), fatty acid transporters (FABP4, CD36), fibroblast-related markers (COL1A2 and PREF1), and neuroreceptor-related markers (CHRM3, CHRM2, ADRB3) using real-time PCR. High-quality electrocardiograms (ECGs) from 100 patients were analysed for 36 ventricular depolarization and repolarization parameters. Principal component analysis was used to explore the internal ECG structure. Spearman correlation with Benjamini-Hochberg correction and sex-stratified linear regression assessed ECG-EAT associations, adjusting for clinical covariates.

**Results:**

In females, EAT fibrotic and inflammatory profile were correlated with delayed late anteroseptal depolarization (prolonged S-wave upstroke time) in V2 (COL1A2, *p*= 0.642, adj.*p* = 0.025; DEFA3, *p* = 0.572, adj.*p* = 0.038), and V3 (COL1A2, *p* = 0.592; DEFA3, *p* = 0.629; CD16, *p* = 0.642; CD14, *p* = 0.596; CHRM3, *p* = 0.600; all adj.*p* ≤ 0.027). Linear regression models confirmed strong positive associations between the interaction ECGxSex and CD14, CD16, DEFA3, and COL1A2 (*β* = 1 to 5, all *p* ≤ 0.02). Clinical factors reduced but did not eliminate sex dependent effects. Akaike information criteria (AIC)-refined models identified coronary artery disease, hypertension, and valvular heart disease as key covariates, yet inflammatory markers (CD14 and CD16) retained sex-specific explanatory power (adjusted *R*^2^ = 0.13–0.20).

**Conclusion:**

Despite similar absolute ECG values, females exhibited a stronger electrical response to the EAT inflammatory profile. These findings highlight inflammation-driven sex-specific depolarization alterations and support ECG as a simple tool to indicate adverse EAT phenotypes.

## Graphical Abstract

**Figure:**
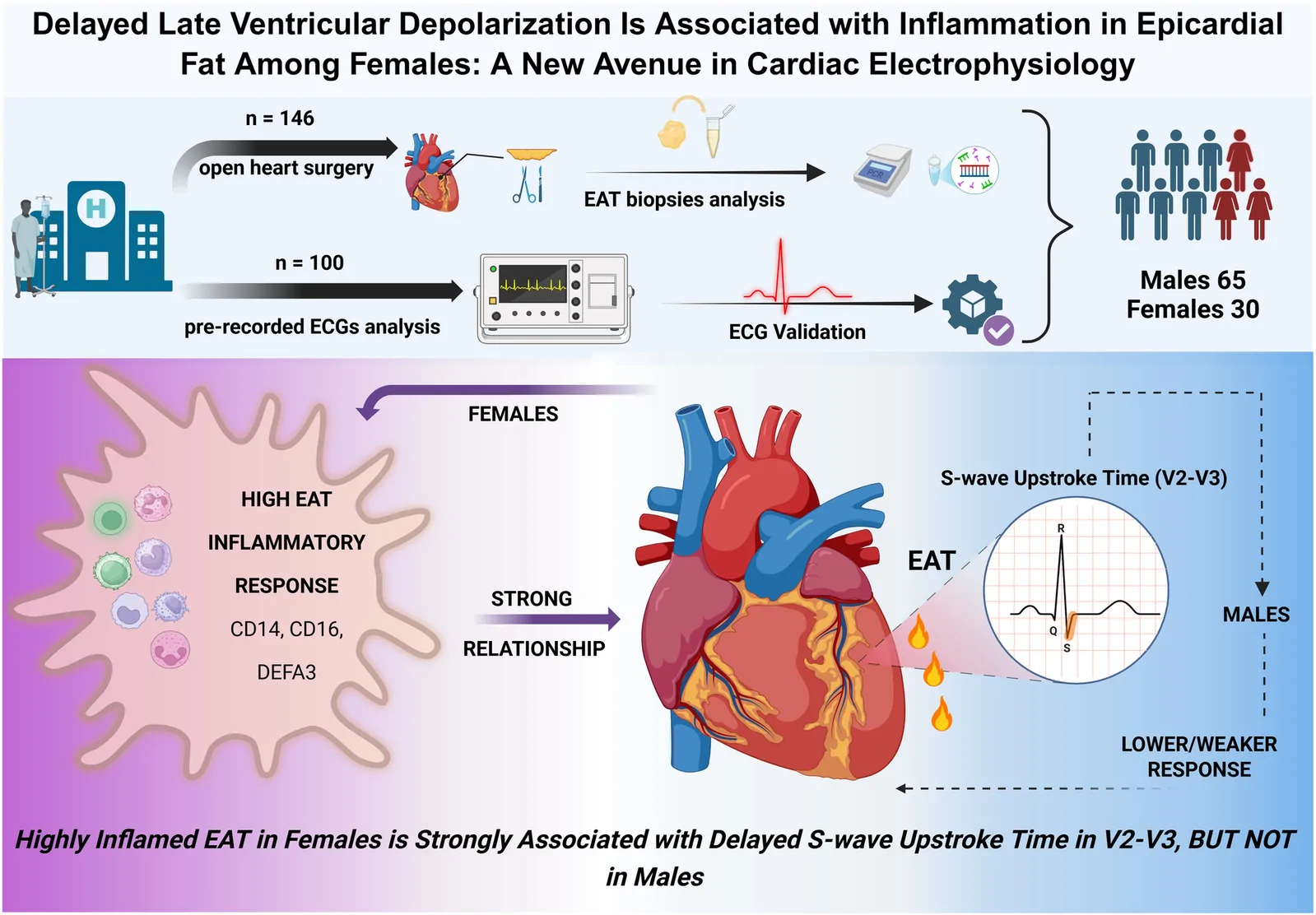

## 1 Introduction

Epicardial adipose tissue (EAT) is a visceral fat depot located between the myocardium and the visceral pericardium, sharing microcirculation with the heart and lacking a fascial barrier, which permits direct cellular and biochemical interactions between adipocytes and cardiomyocytes (1, 2). This unique anatomical proximity enables EAT to modify myocardial structure, coronary artery function, and cardiac electrophysiology through its metabolically active and endocrine properties (2, 3) Clinically, EAT has been consistently linked to arrhythmias, coronary artery disease (CAD), and heart failure (HF). Several studies have demonstrated an association between EAT volume with higher incidence, duration, and recurrence of atrial fibrillation (AF) (4), with peri-atrial EAT influencing atrial remodelling via exosomal signalling, including RNAs (2, 5), and being associated with stroke risk and CHA2DS2-VASc scores in non-valvular AF (6–9). Additionally, EAT on the ventricular free walls is related to premature ventricular arrhythmias in HF patients (10, 11). Thus, EAT could increasingly alter the cardiac electrophysiology and be a potential predictor of arrhythmic risk. Multiple studies have reported connections between EAT volume and myocardial electrical activity, including associations with QRS duration and fragmentation (12, 13).

**Figure 1:**
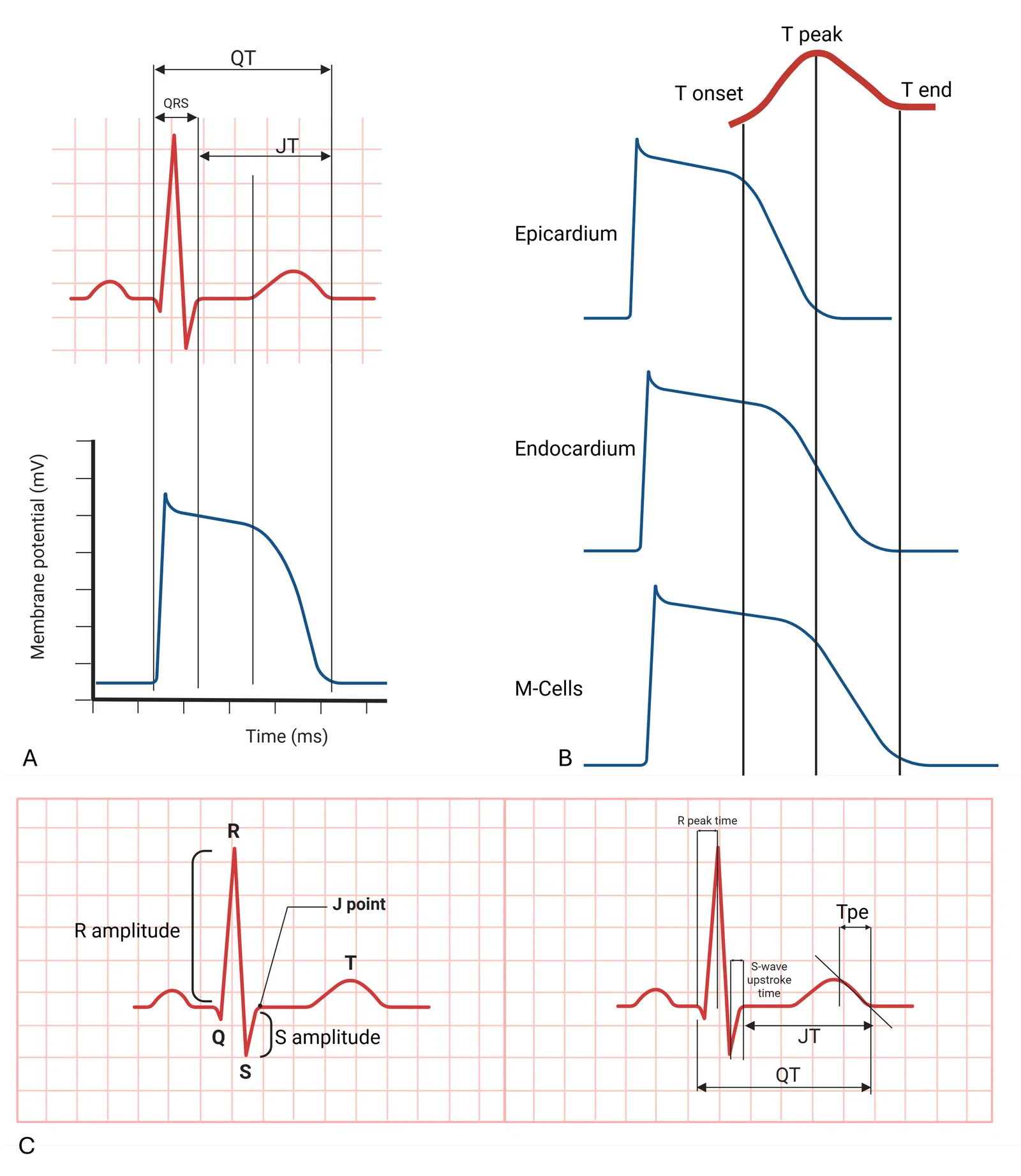
Ventricular electrical activity in ECG. **(A)** QRS duration represents the ventricular depolarization, QT interval represents the ventricular action potential duration, JT interval represents the ventricular repolarization phase. **(B)** Tpe (T peak- T end) represents the repolarization dispersion or the difference in repolarization time across the ventricular wall. Since M cells have the longest repolarization time, T peak corresponds to the end of repolarization in the epicardium, and T end corresponds to the end of repolarization in the M cell. **(C)** ECG parameters scheme showing both depolarization and repolarization parameters used in the study.

Cardiac electrical activity can be divided into depolarization and repolarization phases (Figures 1A,B). Depolarization is assessed primarily from the QRS complex and its components. R-wave peak time, or ventricular activation time (VAT), reflects conduction timing and has been associated with diastolic dysfunction in left precordial leads (14, 15). In eccentric left ventricular hypertrophy, prominent R waves in the right precordial leads and deep Q waves in the left precordial leads may indicate altered septal activation and increased left ventricular mass (15, 16). Terminal activation duration (TAD), or S-wave upstroke time in V1-V3, is a sensitive marker of delayed ventricular depolarization and slow conduction, particularly in arrhythmogenic right ventricular cardiomyopathy (ARVC) (17–19). It may reflect fibrosis or disruption of myocardial bundles and is associated with arrhythmogenic substrate formation. QRS dispersion (QRSd) reflects conduction heterogeneity and has been linked to sudden cardiac death, especially in ARVC (17–19).

Repolarization is evaluated using QT, JT, and Tpeak- Tend (Tpe) intervals. QT dispersion (QTd) reflects spatial differences is repolarization (20). JT and corrected JT (JTc) intervals represent ventricular repolarization duration independent of depolarization, whereas Tpe measures transmural repolarization dispersion.

Since EAT can mimic the myocardial remodelling related to electrophysiological changes, we aimed to investigate the potential association between ventricular conduction and EAT molecular profile, as well as the differential behaviour between males and females.

## 2 Materials and methods

### 2.1 Patient sample

EAT samples were previously collected from 146 patients who underwent open-heart surgery between mid-2020 and mid-2024, with an exclusion criterion of a history of previous cardiac surgery, active severe infection, or cardiogenic shock on admission. All participants provided written informed consent before inclusion. The study followed the principles of the Declaration of Helsinki and received approval from the Galician Clinical Research Ethics Committee (protocol code 2019_439, 22/10/2019).

Clinical information was obtained from electronic health records, including demographic data, medical history, chronic treatments, echocardiographic data, laboratory testing, and electrocardiographic (ECG) records. Echocardiographic assessment included left ventricular ejection fraction (LVEF) calculated by the Simpson biplane method.

### 2.2 Electrocardiogram study

All ECG were recorded during the hospitalization period with a standardized calibration of a speed of 25 mm/s and an amplitude of 10 mm/mV. Precordial leads (V1-V6) were analysed by a single investigator who was blinded to the clinical characteristics at the time of analysis. QRS duration and the QT, QTc, JT, JTc, and Tpe intervals were calculated in all precordial leads, along with the Tpe/QT and Tpe/QTc ratios. The absolute values of these parameters were obtained as the average of each parameter across the precordial leads. R-wave amplitude, S-wave amplitude, R/S amplitude ratio, R-wave peak time and S-wave upstroke time were calculated in V1, V2 and V3. Right-to-left ratios of QRS, QT, QTc, JT, JTc, Tpe, Tpe/QT, and Tpe/QTc were analysed using the formula (V1 + V2 + V3)/(V4 + V5 + V6), and the ratio of the widest QRS in V1 or V2 to the narrowest QRS in V5 or V6. Dispersions of QRS, QTc, JTc, and Tpe were calculated as the difference between the maximum and the minimum values.

QT was measured using the tangent method, as shown in Figure 1C, and corrected using Bazett's formula [QTc = QT/√(RR)]. Tpe was measured from the peak of the T wave to the end of the T wave. JT and JTc were calculated by subtracting QRS from QT and QTc, respectively. R-wave peak time was measured as the interval between the start point of QRS and the tip of the R-wave, considering the tallest peak in the presence of an rR' pattern. S-wave upstroke time was defined as the interval from the deepest point of the S-wave (S-wave nadir) to the end of QRS (J-point). Leads with T waves <1 mm in height were excluded from analyses involving T-wave-dependent parameters.

Ratios among the right precordial leads V1, V2 and V3 to the left precordial leads V4, V5 and V6 were considered to investigate potential local effects of the EAT molecular profile on the cardiac electrophysiology, in addition to the average of each parameter across precordial leads.

Accordingly, we categorised ECG parameters into two main groups. Depolarization parameters included the average precordial QRS duration, QRS dispersion, the QRS ratio between right (V1 + V2 + V3) and left (V4 + V5 + V6) leads, the ratio of the widest QRS in V1 or V2 to the narrowest in V5 or V6, and in V1-V3, R-wave amplitude, S-wave amplitude, R/S amplitude ratio, R-wave peak time, and S-wave upstroke time. Repolarization parameters included the average of precordial QT, QTc, JT, JTc, Tpe, Tpe/QT and Tpe/QTc durations; dispersions of QTc, JTc, and Tpe; and the right (V1 + V2 + V3) -to-left (V4 + V5 + V6) ratios of each parameter (Figure 1C).

We validated the manually measured values against those automatically recorded by the ECG machine using the exact QRS, QT and QTc values.

### 2.3 Tissue and blood sample collection

Small biopsies of epicardial fat (0.2–0.5 g) were obtained during surgery from the upper anterior right ventricular surface near the septum using sterile surgical scissors. All tissue samples were immediately transported to the laboratory and stored at −80 °C until RNA extraction.

### 2.4 RNA extraction and gene expression analysis

Epicardial fat biopsies were lysed, and total RNA was isolated using commercial extraction kits (Qiagen, Hilden, Germany) following the manufacturer's instructions. Complementary DNA (cDNA) was synthesized using the Maxima First Strand cDNA Synthesis Kit (Thermo Fisher Scientific, Waltham, MA, USA). Depending on the protocol, 1–2 µL of cDNA was used for amplification of gene expression. Gene expression was quantified by real-time PCR using FastStart SYBR Green Master (Roche Diagnostic S.L., Barcelona, Spain) or the QuantStudio 3 system (Thermo Fisher Scientific, Waltham, MA, USA). Cycling conditions typically included an initial denaturation at 95 °C for 30 s, followed by 40 amplification cycles at 60 °C for 60 s and 72 °C for 30 s with specific primers. Markers related to fatty acid transporters [CD36, and fatty acid binding protein 4 (FABP4)], inflammatory and immune cells [CD14, CD16, CD68, defensin alpha 3 (DEFA3), and CXC chemokine receptor 2 (CXCR2)], fibroblast-related markers [collagen type I alpha 2 chain (COL1A2), and preadipocyte factor-1 (PREF1)], and neuroreceptors, muscarinic acetylcholine (m3: CHRM3, and m2: CHRM2), and the B3 adrenergic (ADRB3) were analysed according to previously established protocols. Gene expression values were normalized to *β*-actin (ACTB), and relative expression levels were calculated using the antilogarithm of the Ct ratio (Ct gene/Ct ACTB). All the specific primers, forward and reverse, are shown in Supplementary Table S1.

### 2.5 Statistical analysis

All statistical analyses were performed using R software (version 4.5.2) and RStudio (version 2026.01.0). Descriptive analysis: Continuous variables were examined for normality using the Anderson–Darling test. Because most variables showed non-normal distributions, they were summarized using medians and interquartile ranges (IQR), and comparisons between males and females were performed using the Mann–Whitney U test. Categorical variables were presented as frequencies and percentages. Group comparisons were performed using Pearson's chi-square test, and Fisher's exact test was applied when expected cell counts were small. For each categorical variable, sex-specific proportions were calculated to illustrate the distribution of levels within each group.

Principal component analysis (PCA): was performed to examine the internal structure of the ECG depolarization and repolarization parameter sets. Each group was analysed independently because they represent distinct electrophysiological domains. For the depolarization parameters, some variables contained missing values. To avoid excluding subjects and to preserve the correlation structure among markers, PCA-based imputation was applied. The optimal number of dimensions required for imputation was estimated using the (missMDA) package, and missing values were imputed using the (imputePCA) function. PCA was then performed on the completed dataset using the (FactoMineR) and (Factoextra) packages. For both parameter sets, we extracted eigenvalues, variable loadings, squared cosine (cos2) values, contribution indices, and individual component scores. To explore potential sex-related patterns, individual PCA scores were plotted by sex, and 95% confidence ellipses were added to visualize group distribution.

Correlation tests: Spearman's rank correlation coefficients (*ρ*) were calculated to evaluate the association between principal components (PCx) and EAT markers, and subsequently between ECG individual variables and EAT markers. Each analysis was performed in the total population and separately for males and females. To control for the false discovery rate (FDR) due to multiple testing, the Benjamini-Hochberg procedure was applied separately within the total population analysis and within each sex-specific analysis. Adjusted *p*-values (adj.p) corrected using the FDR method were considered statistically significant at *p* < 0.05. ECG-EAT pairs showing preliminary sex-associated differences were selected for further evaluation using regression modelling.

Linear regression model with interaction ECGxSex: To examine more precisely whether the associations between ECGxSex interaction and EAT markers, we performed linear regression models that included an interaction between each selected ECG variable and sex. Because our primary interest was the sex-specific slope of the ECG-EAT relationship, the interaction was modelled using the following form: EAT marker = *β*0 + *β*1 (ECGxSex) + *ε.*

When a significant interaction was detected, sex-stratified models were subsequently fitted to estimate separate slopes and confidence intervals for each sex group. In the second stage, we examined whether individual clinical factors influenced this interaction. For each ECG-EAT pair, every clinical variable was added separately to the interaction model to determine whether it altered the ECG-EAT relationship or modified the interaction term. Each model followed the general structure: EAT marker = *β*0 + *β*1 (ECGxSex) + *β*2 (clinical factor) + *ε.*

This single-covariate approach allowed us to identify which clinical factors attenuated the interaction or affected its precision. Finally, in a third step, we constructed fully adjusted multivariable models that included all the clinical covariates simultaneously. A backward selection procedure based on the Akaike information criterion (AIC) was applied to identify the most efficient model for each ECG-EAT interaction. This approach allowed us to determine whether the sex-dependent ECG-EAT interaction persisted after accounting for the combined influence of all relevant clinical factors.

## 3 Results

### 3.1 Patient sample

**Figure 2:**
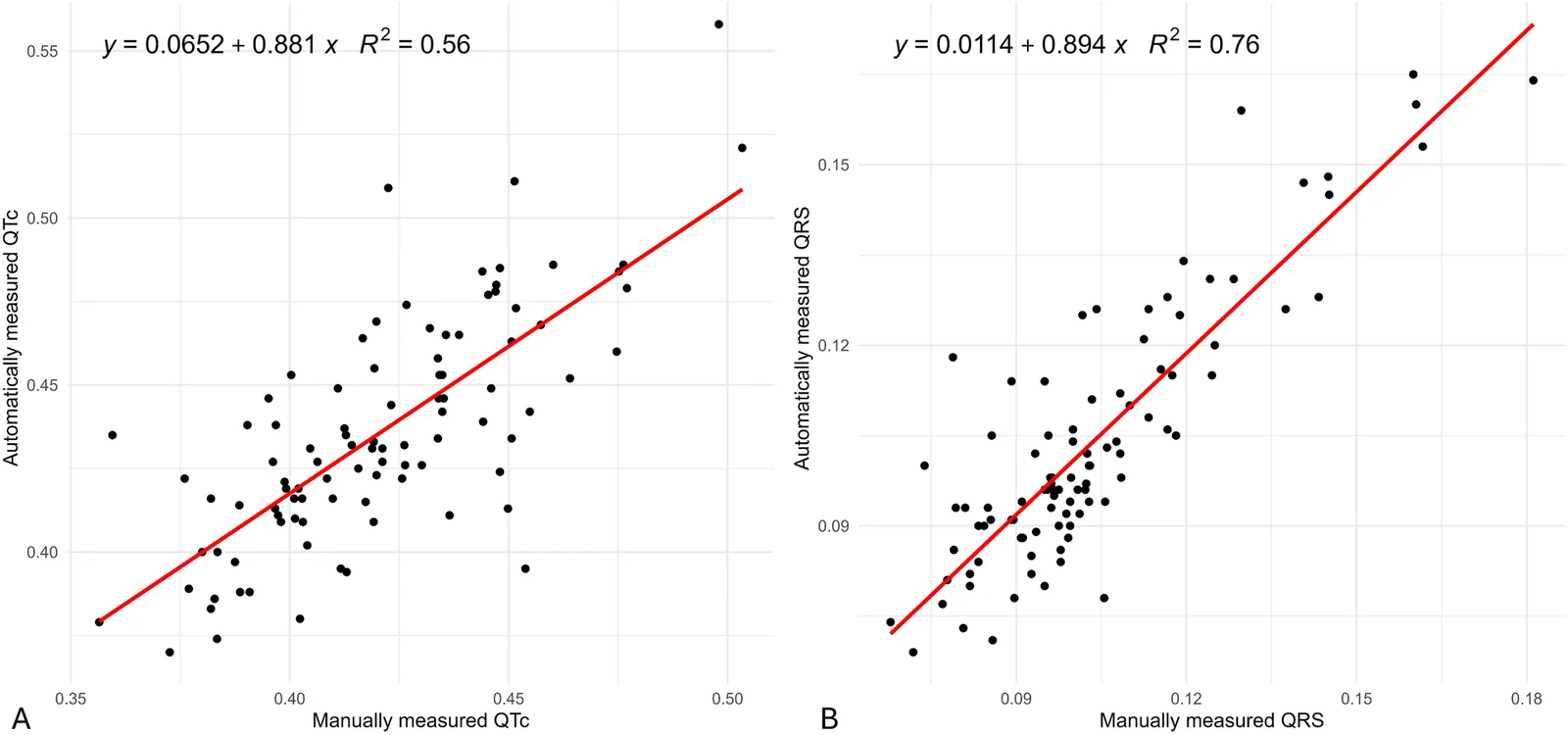
Linear correlations between the manually measured values of QTc **(A)** and QRS **(B)** and the automated values.

**Figure 3:**
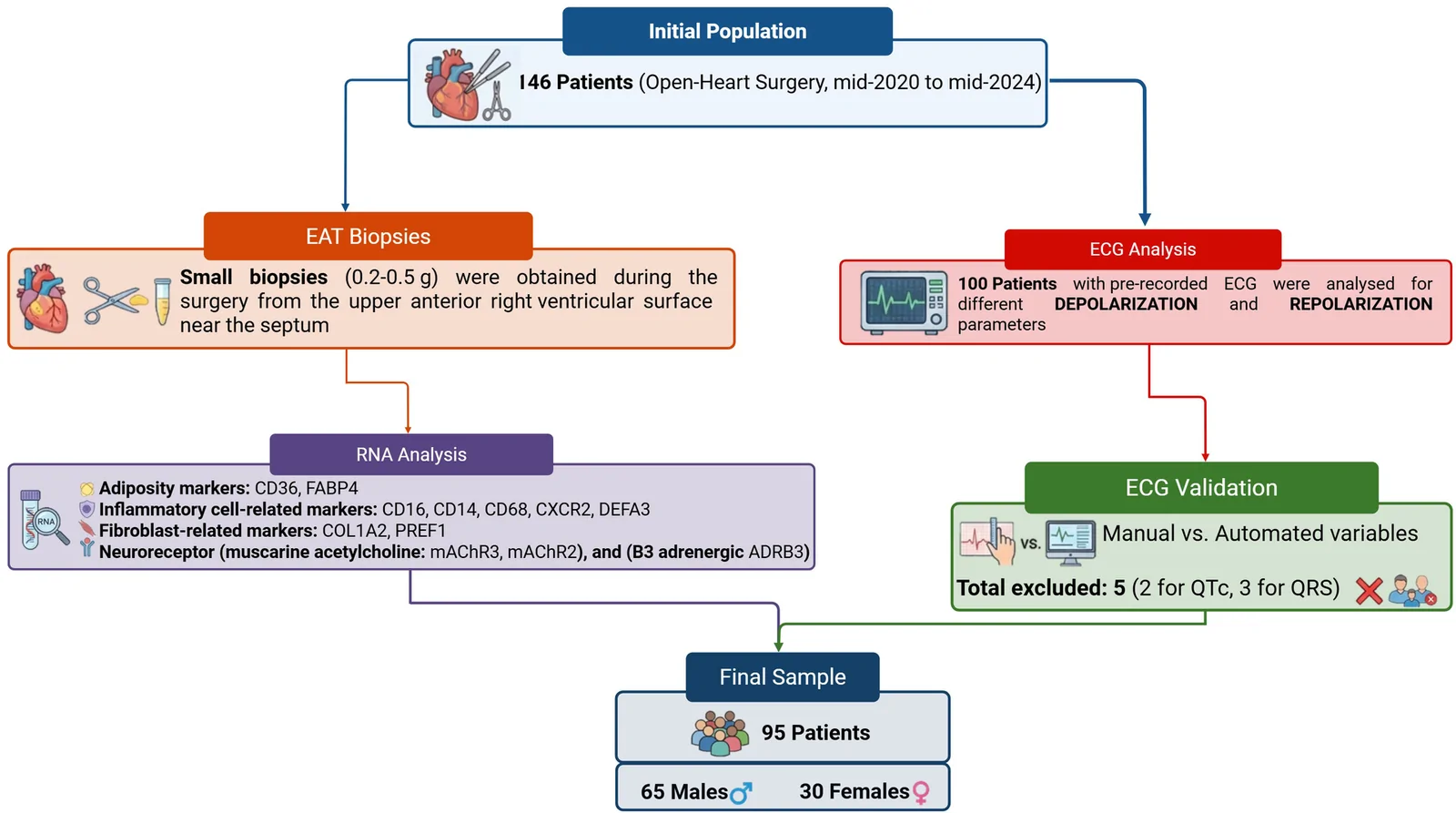
Patient sample inclusion and exclusion criteria. ADRB3, B3 adrenergic receptor; COL1A2, collagen type I alpha 2 chain; CXCR2, CXC chemokine receptor 2; DEFA3, defensin alpha 3; EAT, epicardial adipose tissue; ECG, electrocardiogram; FABP4, fatty acid binding protein 4; mAChR2, M2 muscarinic acetylcholine receptor; mAChR3, M3 muscarinic acetylcholine receptor; PREF1, preadipocyte factor-1.

Out of 146 patients, 100 were analysed for their ECG records before surgery. Using linear correlations between manually measured QRS, QT, and QTc and their automated counterparts, we validated the analysed ECGs. Five ECGs showing marked disagreement between manual and automated measurements were excluded, resulting in 95 ECGs for the final analysis. Two patients were excluded because of QTc adjustment (Figure 2A), and three more were excluded because of QRS adjustment (Figure 2B). The flow chart of the patients included is shown in Figure 3.

After adjusting the ECG parameters, the study population consisted of 95 patients (31.6% females). The most prevalent cardiac conditions were valvular heart disease (VHD) (78.9%), dyslipidaemia (DLP) (71.6%), and hypertension (HTN) (63.2%). While CAD, diabetes mellitus (DM), and HTN were more prevalent in males, VHD, heart failure with preserved ejection fraction (HFpEF), and DLP were more frequent in females. Similarly, regarding electrical disturbances, AF was more common among females, while ventricular and atrioventricular conduction disorders were more prevalent in males. The clinical characteristics are summarized in Table 1.

**Table 1:** Patients’ clinical characteristics.

Variable	Females		Males		Total population		*P* value
	*N*	%	*N*	%	*N*	%	
Gender	30	31.6%	65	68.4%	95		
CAD	11	36.7%	34	52.3%	45	47.4%	0.230
DM	2	6.7%	26	40.0%	28	29.5%	**0.002**
VHD	28	93.3%	47	72.3%	75	78.9%	**0.039**
LVEF > 50%^a^	27	90.0%	47	72.3%	74	78.7%	0.120
HF	13	43.3%	24	36.9%	37	38.9%	0.712
HFpEF	10	33.3%	10	15.4%	20	21.1%	0.084
HTN	16	53.3%	44	67.7%	60	63.2%	0.263
DLP	23	76.7%	45	69.2%	68	71.6%	0.615
AF	8	26.7%	12	18.5%	20	21.1%	0.521
BBB	3	10.0%	9	13.8%	13	13.7%	0.747
AVB	3	10.0%	17	26.2%	20	21.1%	0.127
Tobacco	3	10.0%	24	36.9%	27	28.4%	**0.014**
BB	15	50.0%	43	66.2%	58	61.1%	0.202
Statins	19	63.3%	48	73.8%	67	70.5%	0.422

a Only 94 patients had the values of LVEF%. AF, atrial fibrillation; AVB, atrio-ventricular block; BB, beta blockers; BBB, bundle branch block; CAD, coronary artery disease; DLP, Dyslipidaemia; DM, diabetes mellitus; HF, heart failure; HFpEF, heart failure with preserved ejection fraction; HTN, hypertension; LVEF, left ventricular ejection fraction; VHD, valvular heart disease. *P*-value considers statistical significance between males and females.

*P* value with statistical significance <0.05 is represented in bold number.

The median age was approximately 71 (IQR 63–74) years (Supplementary Table S2) and was significantly higher in females (Table 2). LVEF was significantly lower in males (Table 2). Regarding ECG parameters, the QTc and JTc intervals were longer in females, as well as the dispersion of QTc, JTc, and Tpe. Conversely, QRS and Tpe intervals were longer in males (Supplementary Table S3). However, statistically significant differences were observed only for QRS and JTc (Table 2). All descriptive analyses and sex-related differences are fully described in Supplementary Tables S2, S3.

**Table 2:** Significant differences regarding age, ECG, and LVEF parameters between males and females.

Variable	Males	Females	*P* value
Age (years)	66 (IQR 61–73)	72 (IQR 69–77)	0.011
QRS duration (sec)	0.102 (IQR 0.095–0.116)	0.095 (IQR 0.087–0.102)	0.029
JTc duration (sec)	0.310 (IQR 0.289–0.333)	0.329 (IQR 0.309–0.350)	0.008
LVEF (%)	58 (IQR 50–63)	62 (IQR 56–67)	0.015

IQR, interquartile range; LVEF, left ventricular ejection fraction.

### 3.2 Principal component analysis

PCA was performed for the two groups of ECG variables: depolarization and repolarization variables. In the depolarization group, PCA of the 19 ECG-derived depolarization variables identified four principal components with eigenvalues greater than 1, together explaining 68.922% of the total variance. PC1 accounted for 26.901% of the variance and represented late anteroseptal and right-sided depolarization with an excellent representation of S-wave upstroke time in V2 and V3 (Cos2 ≈ 0.7) (Table 3), followed by PC2 (21.918%, early right ventricular depolarization), PC3 (10.953%, depolarization heterogeneity), and PC4 (9.151%, early anteroseptal depolarization. In a similar way in the repolarization group, PCA of the 17 repolarization-derived ECG variables identified three principal components collectively explaining 62.119% of the total variance. PC1 accounted for the largest proportion of variance (29.989%) and represented regional repolarization ratios, followed by PC2 (18.115%, transmural-related repolarization dispersion) and PC3 (14.015%, corrected repolarization durations and dispersions).

**Table 3:** Highest loading variables, representation quality (Cos^2^), and contributions for principal components in both depolarization and repolarization group.

Variable	Loading	Cos^2^	Contribution
Depolarization group			
PC1 components			
S3_UT	0.838	0.703	13.754
S2_UT	0.834	0.696	13.618
S2_A	0.729	0.532	10.401
S3_A	0.709	0.502	9.823
R/S_3	−0.686	0.471	9.214
R/S_2	−0.603	0.363	7.109
QRS	0.593	0.352	6.881
R3_A	−0.591	0.350	6.845
PC2 components			
R/S_1	0.889	0.790	18.960
R1_PT	0.841	0.708	16.999
R1_A	0.818	0.668	16.051
R/S_2	0.589	0.347	8.322
R2_A	0.542	0.293	7.042
S1_UT	0.508	0.258	6.205
PC3 components			
QRS_R/L	−0.777	0.655	31.497
WQRS/NQRS	−0.810	0.604	29.002
QRS	0.547	0.299	14.364
QRSd	−0.409	0.168	8.051
PC4 components			
R3_A	0.679	0.461	26.532
S1_A	0.531	0.282	16.209
R/S_3	0.522	0.272	15.645
R2_A	0.483	0.233	13.426
Repolarization group			
PC1 components			
QT_R/L	0.862	0.744	14.586
Tpe_R/L	0.858	0.736	14.444
QTc_R/L	0.843	0.711	13.955
Tpe/QT_R/L	0.810	0.656	12.874
JT_R/L	0.795	0.632	12.406
Tpe/QTc_R/L	0.784	0.614	12.045
JTc_R/L	0.767	0.588	11.532
PC2 components			
Tpe	0.904	0.817	26.519
Tpe/QTc	0.797	0.636	20.652
QT	0.638	0.407	13.222
JT	0.623	0.389	12.622
Tpe/QT	0.591	0.350	11.359
PC3 components			
QTc	0.792	0.627	26.324
JTc	0.701	0.491	20.602
QTcd	0.595	0.354	14.873
JTcd	0.573	0.329	13.793

dTpe, dispersion of T peak—T end; JTc, corrected JT; JTcd, dispersion of corrected JT; JT R/L, JT right (V1 + V2 + V3)-to-left (V4 + V5 + V6) ratio; JTc R/L, corrected JT right (V1 + V2 + V3)-to-left (V4 + V5 + V6) ratio; QRSd, QRS dispersion; QRS R/L, QRS right (V1 + V2 + V3)-to-left (V4 + V5 + V6) ratio; QTc, corrected QT; QTcd, dispersion of corrected QT; QT R/L, QT right (V1 + V2 + V3)-to-left (V4 + V5 + V6) ratio; QTc R/L, corrected QT right (V1 + V2 + V3)-to-left (V4 + V5 + V6) ratio; R1 A, R-wave amplitude in V1; R2 A, R-wave amplitude in V2; R3 A, R-wave amplitude in V3; R1 PT, R-wave peak time in V1; R2 PT, R-wave peak time in V2; R3 PT, R-wave peak time in V3; R/S 1, R/S amplitude ratio in V1; R/S 2, R/S amplitude ratio in V2; R/S 3, R/S amplitude ratio in V3; S1 A, S-wave amplitude in V1; S2 A, S-wave amplitude in V2; S3 A, S-wave amplitude in V3; S1 UT, S-wave upstroke time in V1; S2 UT, S-wave upstroke time in V2; S3 UT, S-wave upstroke time in V3; Tpe, T peak—T end; Tpe/QT R/L, Tpe/QT right (V1 + V2 + V3)-to-left (V4 + V5 + V6) ratio; Tpe/QTc R/L, Tpe/QTc right (V1 + V2 + V3)-to-left (V4 + V5 + V6) ratio; Tpe R/L, Tpe right (V1 + V2 + V3)-to-left (V4 + V5 + V6) ratio; WQRS/NQRS, the widest QRS (in V1 or V2)/the narrowest QRS (in V5 or V6).

**Figure 4:**
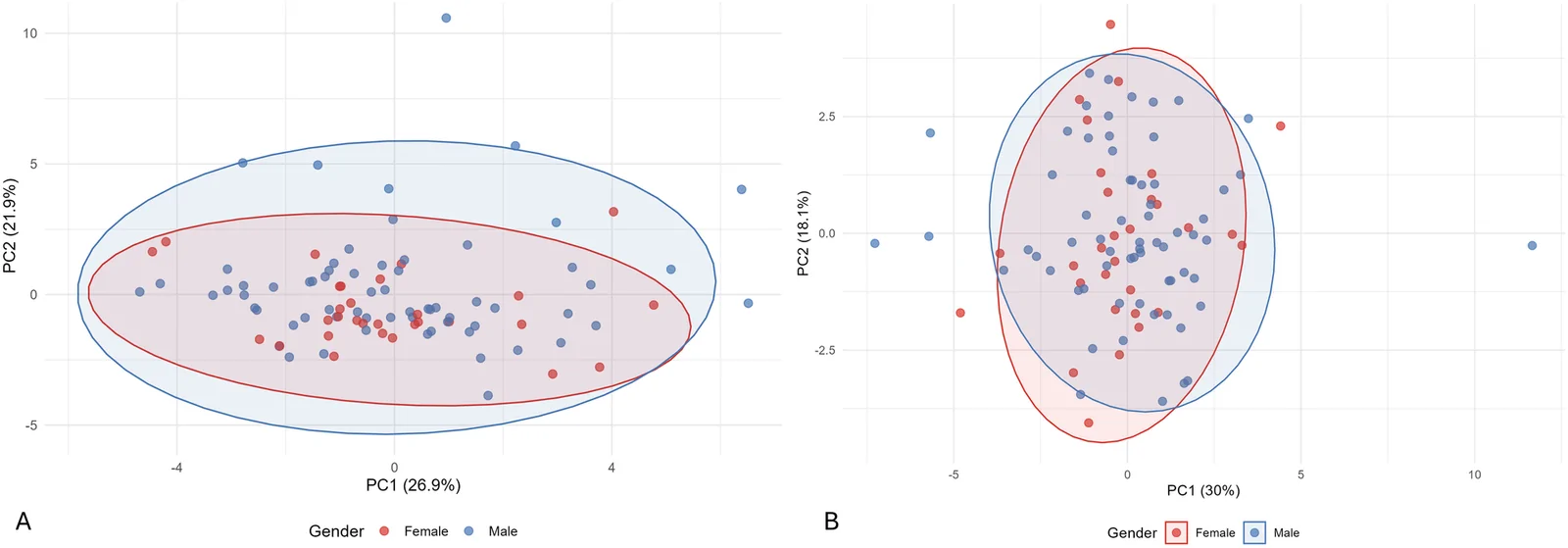
PCA space markers by sex [**(A)** depolarization, **(B)** repolarization]. Data points are coloured by sex. Ellipses represent the 95% confidence regions for each group. PC1 and PC2 define the two-dimensional PCA space summarizing the major patterns of variation in markers.

Visual projection of the sex-based distribution of the variables in the PCA space did not clearly separate males and females, neither in depolarization PC groups nor in repolarization ones (Figure 4). The whole statistical process of PCA is fully described in the Supplementary Data (Supplementary Table S4, Figures S1–S4).

#### 3.2.1 PCA as a validation tool

PCA was also used as an additional validation step for the ECG dataset. By examining how variables were positioned across the principal component space, we observed coherent and physiologically consistent grouping patterns. The clustering of related variables along shared PCA dimensions indicated that the overall structure of the data was consistent with expected electrophysiological relationships.

### 3.3 Associations between PCA components and EAT markers

In depolarization group, correlation analysis between the PCA components and EAT molecular profile markers showed that PC1 was the only component demonstrating significant associations in the total population. Higher PC1 scores were associated with lower expression of fatty acid transporters-related markers (FABP4: *ρ* = −0.256, *p* = 0.012; CD36: *ρ* = −0.230, *p* = 0.025) and higher levels of inflammatory cell-related markers (CD14: *ρ* = 0.219, *p* = 0.033; CD16: *ρ* = 0.210, *p* = 0.046). No significant correlations were observed for PC2, PC3, or PC4 in the total population, indicating that PC1 represents the primary depolarization electrophysiological axis linked to EAT (Supplementary Table S5). Sex-stratified analysis revealed that females demonstrated a strong pattern of correlations, almost exclusively involving PC1. Higher PC1 values were also associated with neuroreceptor-related markers (ADRB3: *ρ* = 0.430, *p* = 0.018; CHRM3: *ρ* = 0.498, *p* = 0.006; CHRM2: *ρ* = 0.439, *p* = 0.016), inflammatory cell-related markers (CD16: *ρ* = 0.533, *p* = 0.003; DEFA3: *ρ* = 0.520, *p* = 0.004; CXCR2: *ρ* = 0.395, *p* = 0.035; CD14: *ρ* = 0.444, *p* = 0.015), and fibroblast-related marker (COL1A2: *ρ* = 0.498, *p* = 0.006). However, none of these correlations remained statistically significant after FDR correction (Supplementary Table S5). Similar results were found for repolarization group, where some significant correlations were observed between PC1 and some EAT markers, particularly in females (CXCR2: *ρ* = 0.448, *p* = 0.016; CD36: *ρ* = −0.402, *p* = 0.028; CHRM3: *ρ* = 0.377, *p* = 0.041; ADRB2: *ρ* = 0.364, *p* = 0.049); again, none of them remained statistically significant after FDR correction.

### 3.4 Associations between ECG depolarization variables and EAT markers

**Figure 5:**
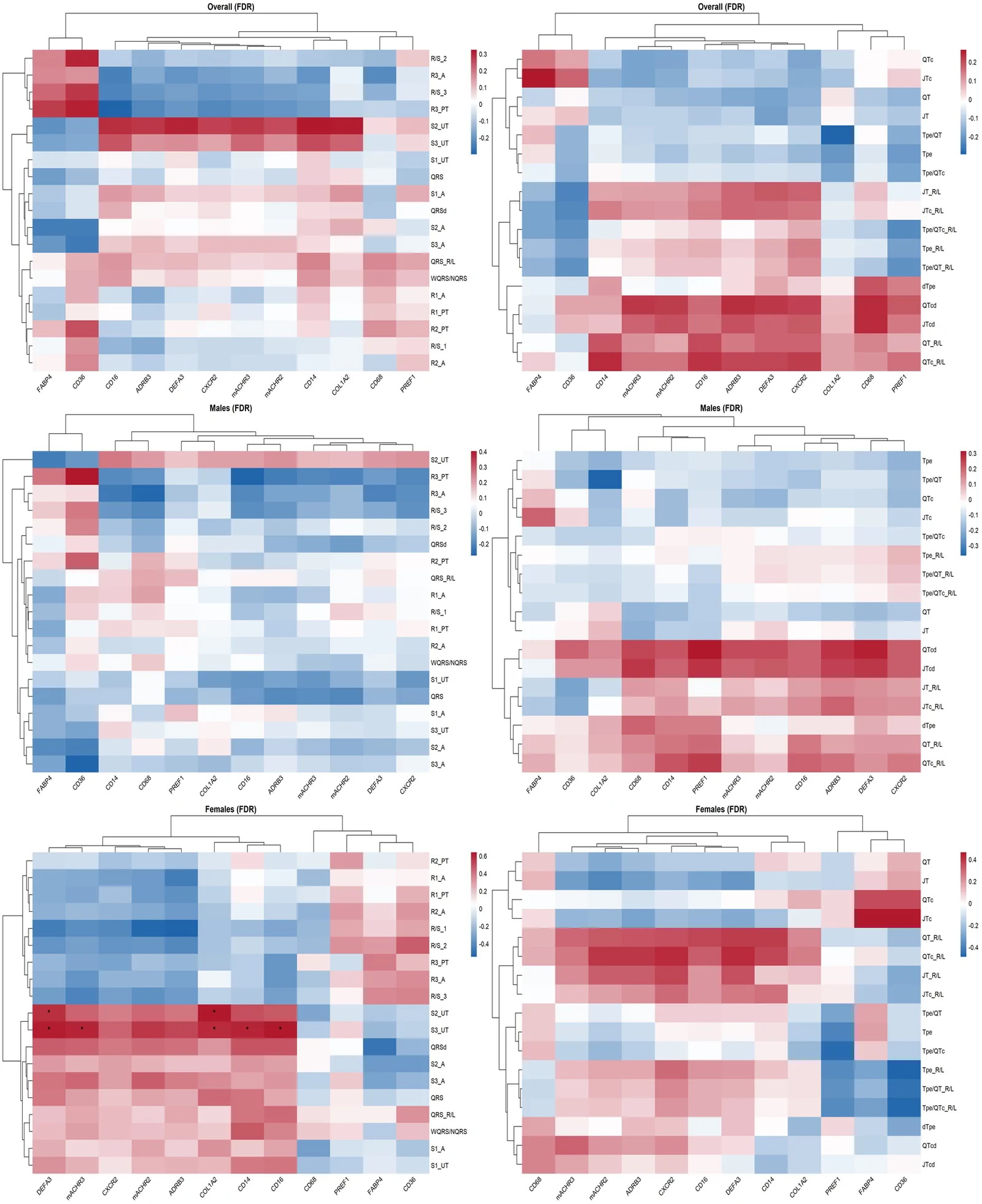
Clustered heatmaps of ECG-EAT correlations in the total population and by sex after FDR correction. Each panel displays a clustered heatmap of Spearman correlation coefficients between EAT markers and ECG depolarization parameters (left panels) or repolarization parameters (right panel), after FDR adjustment for multiple testing, shown for total population (top raw), males (middle row), and females (bottom raw). Asterisks indicate statistically significant associations. ADRB3, B3 adrenergic receptor; COL1A2, collagen type I alpha 2 chain; CXCR2, CXC chemokine receptor 2; DEFA3, defensin alpha 3; dTpe, dispersion of T peak—T end; FABP4, fatty acid binding protein 4; JTc, corrected JT; JTcd, dispersion of corrected JT; JT R/L, JT right (V1 + V2 + V3)-to-left (V4 + V5 + V6) ratio; JTc R/L, corrected JT right (V1 + V2 + V3)-to-left (V4 + V5 + V6) ratio; mAChR2, M2 muscarinic acetylcholine receptor; mAChR3, M3 muscarinic acetylcholine receptor; PREF1, preadipocyte factor-1; QRSd, QRS dispersion; QRS R/L, QRS right (V1 + V2 + V3)-to-left (V4 + V5 + V6) ratio; QTc, corrected QT; QTcd, dispersion of corrected QT; QT R/L, QT right (V1 + V2 + V3)-to-left (V4 + V5 + V6) ratio; QTc R/L, corrected QT right (V1 + V2 + V3)-to-left (V4 + V5 + V6) ratio; R1 A, R-wave amplitude in V1; R2 A, R-wave amplitude in V2; R3 A, R-wave amplitude in V3; R1 PT, R-wave peak time in V1; R2 PT, R-wave peak time in V2; R3 PT, R-wave peak time in V3; R/S 1, R/S amplitude ratio in V1; R/S 2, R/S amplitude ratio in V2; R/S 3, R/S amplitude ratio in V3; S1 A, S-wave amplitude in V1; S2 A, S-wave amplitude in V2; S3 A, S-wave amplitude in V3; S1 UT, S-wave upstroke time in V1; S2 UT, S-wave upstroke time in V2; S3 UT, S-wave upstroke time in V3; Tpe, T peak—T end; Tpe/QT R/L, Tpe/QT right (V1 + V2 + V3)-to-left (V4 + V5 + V6) ratio; Tpe/QTc R/L, Tpe/QTc right (V1 + V2 + V3)-to-left (V4 + V5 + V6) ratio; Tpe R/L, Tpe right (V1 + V2 + V3)-to-left (V4 + V5 + V6) ratio; WQRS/NQRS, the widest QRS (in V1 or V2)/the narrowest QRS (in V5 or V6).

Subsequently, the associations between individual ECG parameters and EAT markers were examined separately. Spearman's rank correlation analysis identified several statistically significant but weak correlations between EAT markers and selected depolarization variables. However, these associations did not remain statistically significant after false discovery rate (FDR) correction in the total population or in males. In contrast, the female subgroup showed a distinct cluster of robust correlations that remained significant after FDR correction, particularly involving S-wave upstroke time in leads V2 and V3 (S2 UT and S3 UT, respectively). Both ECG parameters demonstrated strong positive correlations with several cell-related EAT markers, suggesting a sex-specific pattern of association between ventricular depolarization and EAT-related cellular markers. S2 UT correlated with COL1A2 (*ρ* = 0.642, *p* < 0.001, adj.*p* = 0.025), and DEFA3 (*ρ* = 0.572, *p* = 0.001, adj.*p* = 0.038). S3 UT showed similarly strong associations with CD16 (*ρ* = 0.642, *p* < 0.001, adj.*p* = 0.025), DEFA3 (*ρ* = 0.629, *p* < 0.001, adj.*p* = 0.025), CD14 (*ρ* = 0.596, *p* < 0.001, adj.*p* = 0.027), CHRM3 (*ρ* = 0.600, *p* < 0.001, adj.*p* = 0.027), and COL1A2 (*ρ* = 0.592, *p* < 0.001, adj.*p* = 0.027) (Figure 5).

For the correlations between repolarization variables and EAT markers, several associations were initially identified, with generally stronger correlation coefficients observed in females than in males or in the total population (Supplementary Table S6). However, none of these associations remained statistically significant after FDR correction (Supplementary Table S6; Figure 5). This pattern contrasted with the depolarization variables, for which several ECG–EAT associations remained significant after correction, particularly among females.

### 3.5 Sex differences in ECG-EAT association

To further evaluate the sex differences identified in the correlation analyses, linear regression models were fitted for each selected ECG–EAT pair, including the ECG  ×  sex interaction term. The analyses focused on the depolarization variables S2 UT and S3 UT and the EAT markers CD14, CD16, DEFA3, COL1A2, and CHRM3.

In stratified analysis, female ECG slopes were consistently strong, positive, and statistically significant, whereas male slopes were uniformly weak and non-significant, demonstrating a clear sex-related variation in ECG-EAT coupling.

In S2 UT, stratified models revealed robust positive associations in females for all markers CD14 (*β* = 1.881, 95% CI: 0.722–3.040, *p* = 0.002), COL1A2 (*β* = 1.255, 95% CI: 0.261–2.284, *p* = 0.015), DEFA3 (*β* = 5.088, 95% CI: 2.116–8.060, *p* = 0.002), CD16 (*β* = 3.396, 95% CI: 1.492–5.299, *p* = 0.001), and CHRM3 (*β* = 3.992, 95% CI: 1.577–6.407, *p* = 0.002). In contrast, none of these models reached statistical significance in males: CD14 (*β* = 0.264, 95% CI: −0.419–0.95, *p* = 0.443), COL1A2 (*β* = 0.396, 95% CI: −0.211–1.003, *p* = 0.197), DEFA3 (*β* = 0.894, 95% CI: −1.038–2.826, *p* = 0.358), CD16 (*β* = 0.516, 95% CI: −0.677–1.709, *p* = 0.390), and CHRM3 (*β* = 0.505, 95% CI: −1.089–2.100, *p* = 0.528), (Supplementary Table S7).

A similar pattern was observed for S3 UT models where sex-stratified models also showed strong association in females: CD14 (*β* = 1.835, 95% CI: 0.811–2.858, *p* = 0.001), COL1A2 (*β* = 1.192, 95% CI: 0.288–2.096, *p* = 0.012), CD16 (*β* = 3.390, 95% CI: 1.799–4.982, *p* < 0.001), DEFA3 (*β* = 4.886, 95% CI: 2.338–7.433, *p* < 0.001), and CHRM3 (*β* = 4.131, 95% CI: 2.089–6.173, *p* < 0.001). However, none of these models reached statistical significance in males: CD14 (*β* = 0.062, 95% CI: −0.693–0.817, *p* = 0.870), COL1A2 (*β* = 0.122, 95% CI: −0.524–0.768, *p* = 0.708), DEFA3 (*β* = −0.259, 95% CI: −2.411–1.892, *p* = 0.810), CD16 (*β* = −0.041, 95% CI: −1.406–1.324, *p* = 0.952), and CHRM3 (*β* = 0.002, 95% CI: −1.769–1.772, *p* = 0.999), (Supplementary Table S7).

For repolarization variables-EAT markers correlations, several notable associations were initially observed, and these correlations appeared stronger in females than in males or in the total population (Supplementary Table S6). However, none of these associations remained statistically significant after applying FDR correction (Supplementary Table S6, Figure 5), in contrast to the depolarization variables-EAT markers associations.

For the correlations between repolarization variables and EAT markers, several notable associations were initially observed, with generally stronger correlations in females than in males or in the total population (Supplementary Table S6). However, none of these associations remained statistically significant after FDR correction (Supplementary Table S6, Figure 5), in contrast to the associations observed between depolarization variables and EAT markers.

For the correlations between repolarization variables and EAT markers, several associations were initially identified, with generally stronger correlation coefficients observed in females than in males or in the total population (Supplementary Table S6). However, none of these associations remained statistically significant after FDR correction (Supplementary Table S6; Figure 5). This pattern contrasted with the depolarization variables, for which several ECG–EAT associations remained significant after correction, particularly among females.

In the combined-sex models, significant ECG  ×  sex interactions were observed for 7 of the 10 ECG–EAT pairs, including all five S2 UT associations and three S3 UT associations (CD14, CD16, and COL1A2), indicating different behaviour in males and females according to ECG–EAT slopes. Three additional interactions, all involving S2 UT (CD16, COL1A2, and DEFA3), showed a direction consistent with a relatively stronger association in males. However, the corresponding male-specific slopes were not statistically significant. Therefore, the direction of the interaction should be interpreted as a relative difference in slopes between sexes, rather than as evidence of a significant positive ECG–EAT association in males.

In the combined-sex models, significant ECG  ×  sex interactions were observed for 7 of the 10 ECG–EAT pairs, including all five S2 UT associations and three S3 UT associations (CD14, CD16, and COL1A2), indicating significant differences in the ECG–EAT slopes in females. Three additional interactions, all involving S2 UT (CD16, COL1A2, and DEFA3), were oriented toward a relatively stronger association in males. However, the corresponding male-specific slopes were not statistically significant. Thus, the direction of the interaction should be interpreted as a relatively different behaviour in slopes in males and females, rather than as evidence of a statistically significant ECG–EAT association in males.

**Figure 6:**
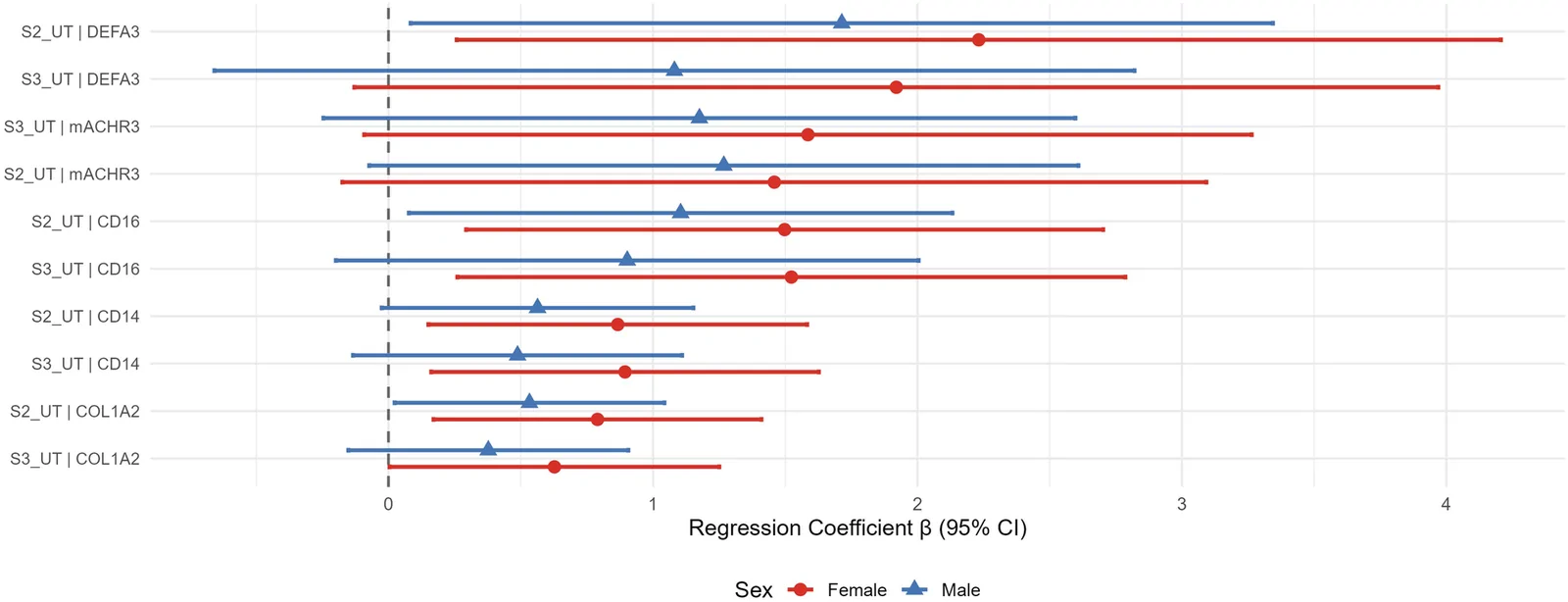
Comparisons of sex-specific ECG-EAT interactions across all ECG-EAT studied pairs. Red circles represent the regression sex-interaction slopes **(b)** and their 95% confidence intervals for females, and blue triangles for males, across all S2 UT and S3 UT associations with EAT markers (CD14, CD16, DEFA3, COL1A2, and mAChR3). COL1A2, collagen type I alpha 2 chain; DEFA3, defensin alpha 3; mAChR3, M3 muscarinic acetylcholine receptor; S2 UT, S-wave upstroke time in V2; S3 UT, S-wave upstroke time in V3.

Overall, the sex-stratified analyses demonstrated a consistent pattern of strong positive ECG–EAT associations in females, with regression coefficients generally ranging from approximately 1 to 5, whereas the corresponding associations in males were weak and statistically non-significant. The formal ECG  ×  sex interaction analyses further supported significant sex differences for most of the evaluated ECG–EAT pairs, particularly for S2 UT and selected S3 UT associations (Figure 6). These findings suggest that the observed sex-related behaviour in ECG–EAT coupling is predominantly characterized by stronger positive associations in females, rather than by significant ECG–EAT associations in males.

### 3.6 Single-covariate regression models assessing the influence of clinical factors on the ECG × Sex interaction

To assess whether individual clinical factors could influence these interactions, we performed single-covariate regression models by adding each of these nine clinical factors (CAD, HTN, VHD, HFpEF, DM, AF, age, BMI, and DLP) one at a time to the interaction model. Across all EAT-ECG pairs, the unadjusted interaction remained significant, and this pattern generally persisted after adjustment.

**Figure 7:**
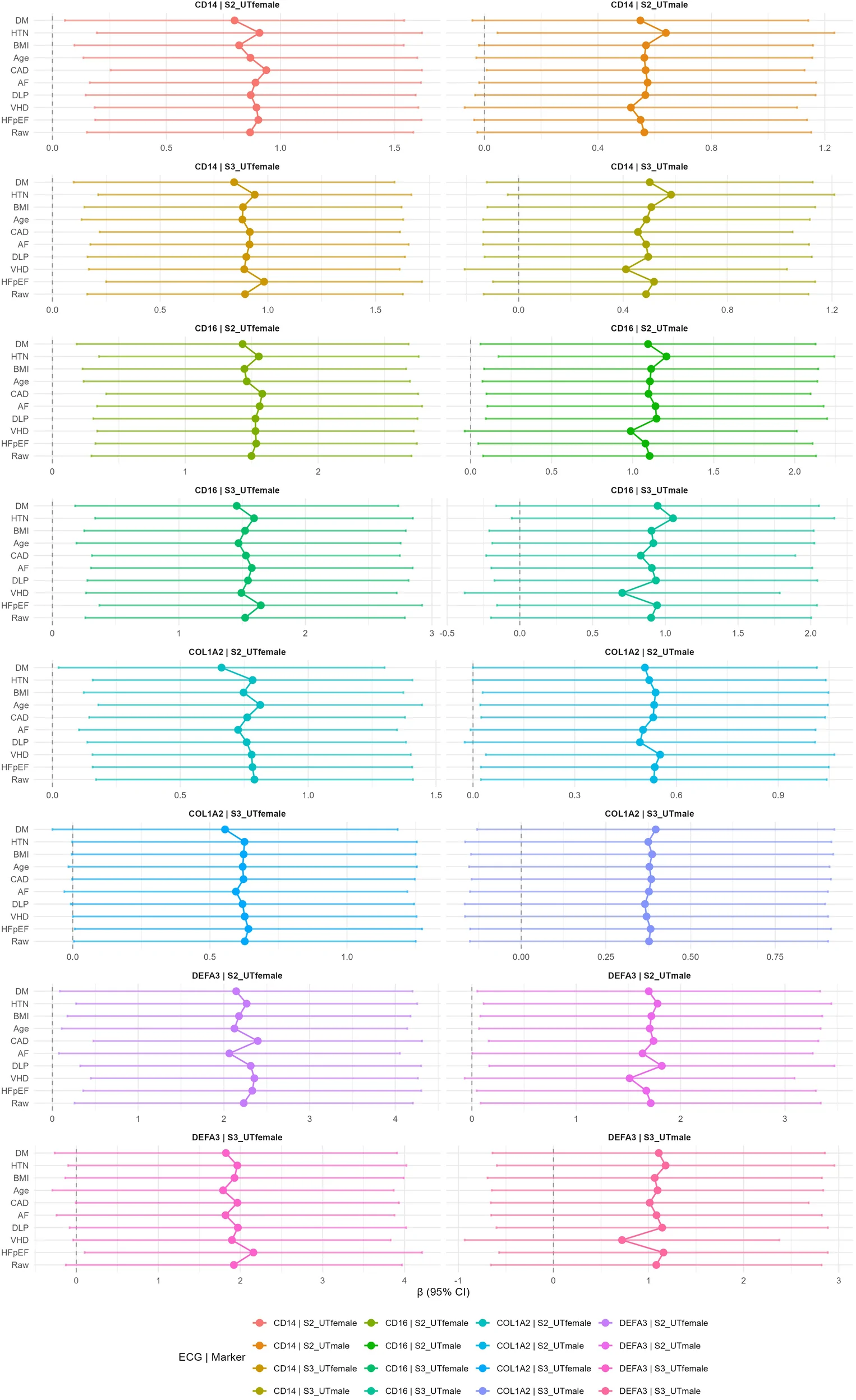
Stability of interaction effects after adding clinical factors. Each horizontal line represents the interaction effect obtained from a single-covariate regression model in which one clinical factor was added to the base interaction model. “Raw” refers to the unadjusted interaction estimated (no clinical factor included). Points represent the shifts in b and reflect the changes in the strength of the sex-dependent interaction, while lines represent the changes in 95% confidence interval width which reflect the differences in the precision of the estimate after adjusting for each clinical factor. AF, atrial fibrillation; BMI, body mass index; CAD, coronary artery disease; DLP, Dyslipidaemia; DM, diabetes mellitus; HFpEF, heart failure with preserved ejection fraction; HTN, hypertension; VHD, valvular heart disease.

**Figure 8:**
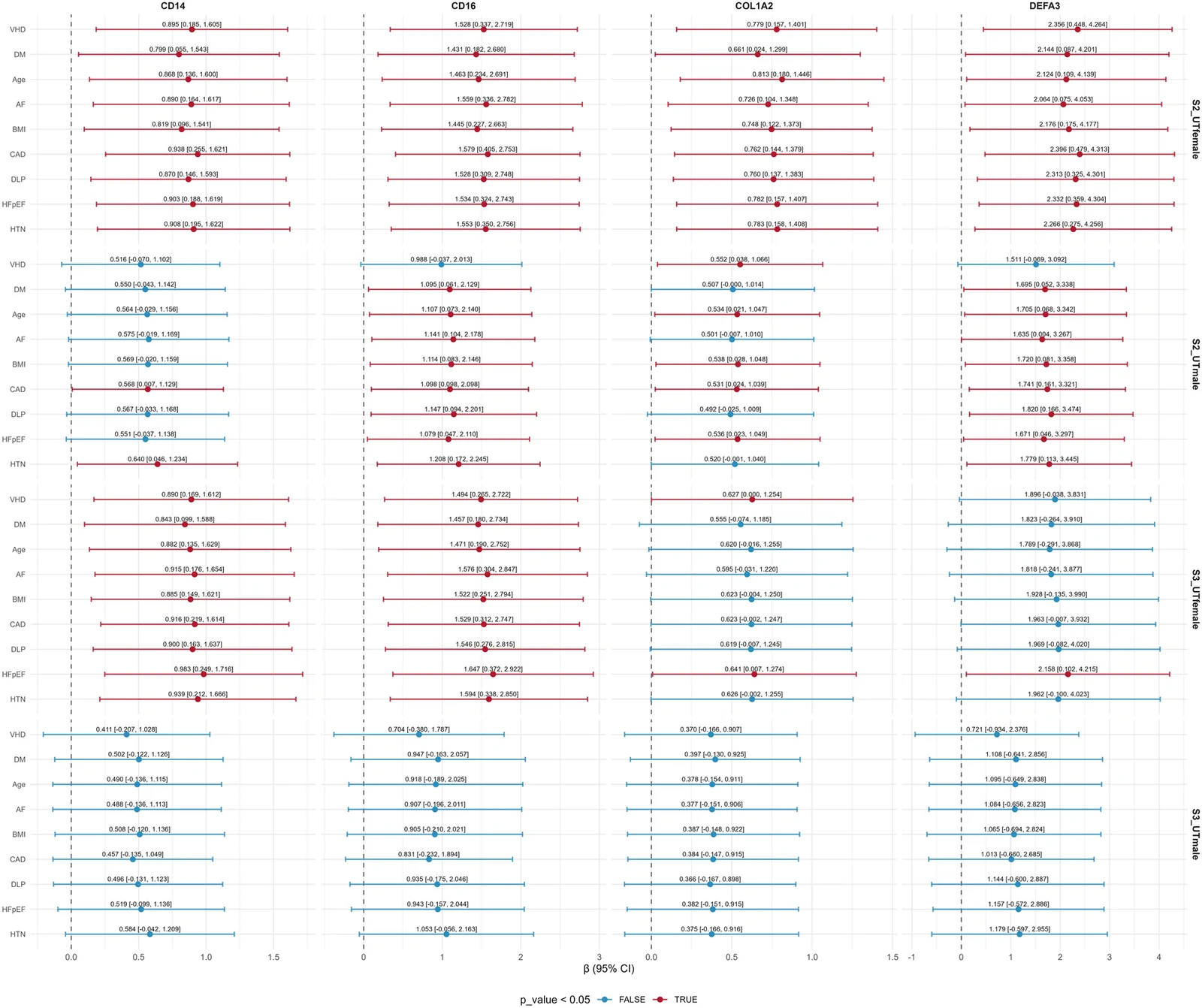
A Forest plot of the estimated interaction coefficients b and their 95% confidence intervals for each ECG × Sex interaction after adding each clinical factor individually to the model. Each horizontal line represents the interaction estimate obtained from a single-covariate regression model. The points indicate the adjusted interaction b, and the horizontal bars represent the 95% confidence intervals. AF, atrial fibrillation; BMI, body mass index; CAD, coronary artery disease; DLP, Dyslipidaemia; DM, diabetes mellitus; HFpEF, heart failure with preserved ejection fraction; HTN, hypertension; VHD, valvular heart disease.

In females, ECG effects remained significant after adjustment for almost every clinical factor, with varying degrees of attenuation across EAT markers. The largest reductions were observed for DEFA3, where effect sizes decreased by approximately 2.5–3 units in both S2 UT and S3 UT models. CD16 showed moderate attenuation (1.7–1.9 units), whereas CD14 and COL1A2 exhibited minimal reductions, typically less than one unit. Confidence intervals narrowed correspondingly, with the greatest narrowing of confidence intervals occurring for DEFA3 and CD16. Notably, these changes were more pronounced in S2 UT than in S3 UT, suggesting that S3 UT associations are more robust to adjustment for individual clinical factors (Supplementary Table S8, Figures 7, 8).

In males, unadjusted models showed no significant ECG-EAT associations. After adjusting for clinical factors, no significant effects were observed in S3 UT after any adjustment. However, significant positive effects emerged in S2 UT only for CD16, DEFA3 and COL1A2, and even when present, male effect sizes were consistently smaller and less precise than female effects (Supplementary Table S8, Figures 7, 8).

### 3.7 Stepwise refinement of interaction models using AIC-based selection

We fitted fully adjusted multivariate models including all nine clinical factors and then applied backward elimination using the AIC to identify the most efficient model for each ECG-EAT pair studied. We observed that CAD, HTN, and VHD were the most consistently associated factors across models, while others were consistently excluded. Importantly, the AIC-selected models revealed three distinct patterns regarding the sex interaction. For CD14 and CD16, both the female and male interaction terms were retained in S2 UT, although the female coefficient remained larger, indicating a persistent sex difference despite a reduced male contribution. In S3 UT, however, only the female interaction term was retained for CD16, reflecting a more consistent and sex-specific pattern in which ECG-EAT coupling was driven exclusively by females. For DEFA3 and COL1A2, the selected models did not retain any sex interaction terms; instead, only clinical factors remained. This aligns with the single-covariate findings, where DEFA3 showed the largest attenuation after adjustment, suggesting that its previously observed sex differences are largely explained by underlying clinical comorbidities.

In the CD14 models, the final AIC-selected structures retained CAD and HTN with AIC values of −282.008 and −296.849 and adjusted *R*^2^ values of 0.182 and 0.204 for S2 UT and S3 UT, respectively. For CD16, the retained associated factors also included CAD and HTN for both S2 UT and S3 UT, yielding final AIC values of −177.664 and −187.242 and adjusted *R*^2^ values of 0.136 and 0.171, respectively. In contrast, both DEFA3 models retained only VHD, with final AIC values of −94.947 and −103.401 and adjusted *R*^2^ values of 0.128 and 0.181 for S2 UT and S3 UT, respectively. Taken together, these results show that sex-dependent ECG-EAT coupling remains robust for CD14 and CD16, particularly in S3 UT, whereas DEFA3 and COL1A2 lose their sex-specific structure after accounting for clinical factors (Table 4).

**Table 4:** Stepwise refinement of interaction models based on AIC.

ECG	Marker	AIC full	AIC final	*R* ^2^	Adj.*R*^2^	Retained variables
S2_UT	CD14	−270.648	−282.008	0.220	0.182	CAD1, S2_UT:Sex_Female, HTN1, S2_UT:Sex_Male
S2_UT	CD16	−165.380	−177.664	0.177	0.136	S2_UT:Sex_Female, CAD1, S2_UT: Sex_Male, HTN1
S2_UT	DEFA3	−88.085	−94.947	0.168	0.128	VHD1, DM1
S3_UT	CD14	−286.515	−296.849	0.248	0.204	CAD1, S3_UT: Sex_Female, HTN1, S3_UT: Sex_Male
S3_UT	CD16	−178.756	−187.242	0.218	0.171	S3_UT: Sex_Female, HTN1, CAD1
S3_UT	DEFA3	−97.976	−103.401	0.235	0.181	VHD1

CAD, coronary artery disease; DEFA3, defensin alpha 3; DM, diabetes mellitus; HTN, hypertension; S2 UT, S-wave upstroke time in V2; S3 UT, S-wave upstroke time in V3; VHD, valvular heart disease.

## 4 Discussion

These original results showed that: 1) The inflammatory EAT profile is associated with cardiac electrical activity, specifically a delay in late depolarization, represented by a prolongation of the S-wave upstroke time in V2-V3; 2) these associations are stronger in females; 3) although cardiac electrical activity is similar between males and females, EAT inflammatory markers differentially associated with this electrical activity.

Across all analyses, the most pronounced sex-specific pattern emerged from the depolarization measures of S-wave upstroke time in V2 and V3, with the value in V3 providing the most stable, discriminating signal. Females consistently showed stronger ECG-EAT associations than males, and this difference was most evident for the chronic inflammatory markers CD14 and CD16 within EAT. These associations remained robust across unadjusted models, single-covariate adjustments, and AIC-selected multivariable structures, indicating a stable and reproducible female-prominent pattern. In contrast, DEFA3, a marker of acute neutrophil-mediated inflammation, showed weaker and less consistent sex-related differences after full adjustment, while COL1A2 contributed minimally to model performance. Taken together, these findings suggest that S-wave upstroke time in leads V2–V3, particularly in V3, may represent a potential ECG correlate of inflammatory cell infiltration in EAT among females.

To our knowledge, this study is the first to show an association between the molecular composition of EAT and specific ECG parameters. Our findings reveal a clear association between the inflammatory profile of EAT and distinct ECG conduction abnormalities, suggesting that the ECG may represent a potential research marker associated with a higher-risk EAT profile. Whereas prior work has proposed imaging-based measures of EAT volume, atrial size, and coronary inflammation for non-invasive AF risk stratification (21), our results suggest that the more widely available and lower-cost ECG may be associated with the same underlying inflamed EAT substrate at the level of ventricular conduction. These associations were consistently stronger in females, suggesting potential sex-related differences in the association between EAT inflammation and ECG parameters. Further studies are needed to explore the potential utility of ECG as a non-invasive tool for the EAT inflammatory profile; if confirmed, ECG could be useful for patient follow-up and for the assessment of potential treatment responses.

### 4.1 ECG parameters and EAT composition

Unsurprisingly, inflammatory cell-related markers showed the strongest associations with ECG parameters. This process was associated with late anteroseptal depolarization, represented by a prolonged S-wave upstroke time in the right anterior precordial leads V2 and V3. However, regarding repolarization parameters, no meaningful associations were detected.

In a recent study from our group, inflammatory adipogenesis in EAT was shown to increase the activity of Nav1.5 (sodium voltage-gated channel alpha subunit 5) and KCa1.1 (potassium calcium-activated channel subfamily A member 1, also known as BK) in adjacent cardiomyocytes through paracrine signalling (22).

Nav1.5 is the principal cardiac voltage-gated sodium channel responsible for the rapid depolarization (phase 0) of the cardiac AP. Increased activity of this channel enhances the late sodium current, thereby prolonging sodium influx during both phase 2 (plateau phase) and phase 3 of cardiac AP. Consequently, this delays late depolarization and prolongs repolarization and extends overall action potential duration (APD) (23, 24). KCa1.1 contributes to repolarization by coupling membrane depolarization with intracellular calcium influx, modulating excitability particularly under pathological conditions (25). Although this mechanism may appear protective as a non-inflammatory adipogenesis effect, inflammation may be associated with APD prolongation and may influence repolarization dispersion through possible effects on multiple potassium channels, most notably by reducing the transient outward K+ current (Ito) (2). However, we did not observe significant associations between repolarization parameters and EAT molecular profile. It remains unclear whether this is due to a protective role of KCa1.1 or simply the need for a larger sample size to detect more subtle effects.

Although both inflammation and adipogenesis modify calcium dynamics in cardiomyocytes (2), the main outcomes of the adipogenesis process: lipid accumulation and gap junction remodelling, tend to affect conduction velocity more than repolarization (26). Therefore, adipocyte accumulation may be associated with the changes in the QRS complex among anteroseptal leads, likely through possible endocrine effects on the myocardium.

We suggest that the alterations in late ventricular depolarization, reflected by a longer S-wave upstroke time, may reflect associations between inflammatory EAT and ion-channel changes. Since the same study demonstrated that semaglutide suppresses EAT inflammation and normalizes ion channels expression (22), an important clinical question arises: to what extent can the ECG be considered a potential correlate of the severity of inflamed EAT? Furthermore, to what extent could the ECG serve as a research tool for assessing therapeutic efficacy in reducing EAT inflammation?

### 4.2 Sex-related electrophysiological differences

Sex-related differences in cardiac electrophysiology are well documented. In our study, and consistent with prior evidence, QTc and JTc intervals, along with their dispersion, were longer in females, whereas QRS duration was longer in males. However, statistically significant sex-related differences emerged only for JTc and QRS duration. After applying PCA, nearly all components were similar between males and females.

Previous studies have reported lower QRS voltage in females (27, 28), narrower QRS complexes (27, 29), smaller T-wave amplitude with a less steep ascent (27, 30), a slower ST-segment slope (30, 31), and lower QT dispersion (32). At the cellular level, female cardiomyocytes are smaller, contract more slowly, and exhibit longer APs (33). Repolarization variables differ between males and females (34, 35), and only females demonstrate an age-related increase in heart rate and a shortening of the mean RR interval (32, 36, 37), likely due to differences in autonomic tone (38).

Despite these established differences, our findings highlight a more specific pattern: the stronger association between the inflammatory EAT molecular profile and depolarization parameters in V2-V3 among females was confined to late depolarization, reflected by the S-wave upstroke time, rather than early depolarization or total depolarization time (QRS duration). This is noteworthy because QRS duration was the only electrical marker showing a clear sex-related difference. Sex-related differences in the QRS complex arise primarily from variations in the spatial distribution and propagation of ventricular depolarization, rather than differences in the amplitudes or durations of individual R or S waves. Females exhibit a higher proportion of the first orthogonal component of QRS power, resulting in smoother and less complex depolarization pathways, shorter QRS duration, and reduced QRS dispersion (39). Importantly, after adjusting for cardiac size and mass, the amplitudes and durations of individual R and S waves do not differ significantly between males and females (29, 39), indicating that electrophysiological variation stems from mechanisms beyond simple anatomical scaling (39).

S-wave upstroke time (TAD) ≥ 55 milliseconds in any lead of V1-V3 is considered prolonged (in the absence of complete right bundle branch block) (40). Prolonged TAD in V1-V3 helps in differentiating ARVC from idiopathic right ventricular outflow tract tachycardia and can be the sole ECG abnormality in gene-positive family members (40). Although the mean value was less than 50 milliseconds in our sample, our findings suggest that this parameter may merit further investigation as a potential marker for risk stratification and early detection of conduction disease in susceptible individuals.

### 4.3 Sex-specific interactions between EAT inflammation and ventricular conduction

Females tend to mount a more pronounced systemic inflammatory response, including higher cytokine release and greater cortisol elevation (41, 42). Additionally, the relationship between systemic inflammation and ECG manifestations appears to differ by sex (43). However, the associations we observed between ECG variables and EAT inflammatory markers may reflect localized associations with EAT-related electrical activity rather than generalized electrical disturbances.

EAT-derived cytokines and profibrotic mediators can induce localized myocardial fibrosis, gap-junction disruption, and conduction slowing in regions adjacent to inflamed EAT (2, 44, 45). Females, particularly postmenopausal, appear more susceptible to EAT expansion and inflammation, which are linked to concentric remodelling, diastolic dysfunction, and microvascular impairment (46–50). This susceptibility may be associated with the more pronounced conduction slowing and delayed late depolarization observed in females in our study.

Furthermore, the female myocardium may have heightened sensitivity to paracrine inflammatory signalling from EAT, potentially due to hormonal influences and sex-specific differences (46–49). EAT thickness increases more steeply with age in females, and its association with cardiac function and electrical parameters becomes stronger in females over 60 years, whereas in males the relationship is attenuated or absent (51). Mechanistic studies support that EAT can induce conduction delay and regional myocardial fibrosis (2, 45), associations that may be more pronounced in females (50, 51), reinforcing the plausibility of these sex-specific electrophysiological interactions.

Another possible explanation relates to the decline in oestrogen levels after menopause. Oestrogen and progesterone modulate cardiac electrical activity at the cellular level, generating unique electrical features in females compared with males (52–54). Oestrogen also mitigates the electrophysiological consequences of EAT inflammation by reducing inflammatory activity, limiting cytokine release, preserving gap-junction integrity, and reducing fibrosis and conduction slowing (50, 55). Its decline after menopause has been associated with increased EAT accumulation, heightened inflammation, and greater susceptibility to both conduction delay and repolarization abnormalities (49, 50).

Taken together, oestrogen decline is strongly linked to oxidative stress (55, 56), and female cardiomyocytes may be more vulnerable to electrical remodelling in the presence of a highly inflammatory and fibrotic EAT profile (57).

### 4.4 The potential role of different cardiovascular diseases in the sex-specific interactions between EAT inflammation and ventricular conduction

Because EAT surrounds the cardiac walls, it may exert paracrine effects on cardiac function. Inflammatory activity is an independent cardiovascular risk factor associated with increased immune-cell infiltration, particularly macrophages, which correlates with CAD severity, atherosclerosis, HF, and other cardiac conditions (57–59). In the line with this, imaging studies have shown that EAT-related coronary inflammation is elevated in AF patients alongside greater EAT volumes and larger atrial dimensions, with the AF association persisting even in patients with minimal coronary disease (21). Moreover, EAT inflammation itself has been associated with fibrosis and oxidative stress, which may contribute to greater cardiac injury (59–61). Based on our findings, particularly the inflammatory EAT composition adjacent to the anteroseptal wall, we suggest a more localized association, manifesting mainly as slower late conduction.

In our sample, CAD was more prevalent in males, consistent with the literature, yet this did not diminish the unique ECG-EAT associations observed in females in both single and multivariate regression models. Females are more prone to coronary microvascular dysfunction and often exhibit less severe obstructive CAD, potentially preserving the substrate through which EAT-mediated inflammation is associated with electrophysiological influence (46, 62, 63). As a result, EAT-related inflammation may contribute to microvascular dysfunction and ECG abnormalities independently of obstructive CAD in females.

Additionally, although many clinical covariates showed associations with sex-related ECG-EAT interactions in single-covariate regression models, most lost statistical significance in the fully adjusted model. Collectively, these findings underscore the predominant, but not exclusive, pattern role of female-specific myocardial biology and EAT-related remodelling, particularly in the context of different cardiovascular conditions. Thus, both female sex and EAT biological behaviour appear to be the main factors associated with the observed electrical phenotypes.

### 4.5 Limitations

This is a single-centre study with a relatively small sample of patients whose ECGs were recorded under different conditions and at varying time points. ECG acquisition was performed under routine clinical conditions without additional standardization beyond preoperative timing and standard calibration. However, patients with active severe infection or cardiogenic shock were excluded to reduce potential confounding of marker levels. Manual measurements were performed by a single reader without the use of specialised measurement techniques; therefore, we used both the PCA method and linear correlations between the manually and automatically measured values as a form of validation. The relatively small sample and the number of comparisons performed warrant cautions interpretation; the regression findings are exploratory and require validation in larger cohorts. As the composition of EAT had already been pre-analysed, we were unable to establish normal or abnormal ranges for these markers and therefore could not precisely determine their effects. In other words, it was not possible to identify the exact point at which the underlying process began. Menopausal status and hormonal data were not available in this study, and therefore potential hormonal influences on the observed female-predominant ECG-EAT associations could not be assessed. Finally, although we found statistically significant relationships, we could not provide a strong clinically explanation due to the small sample size. We still require larger samples to validate these results and to identify alternative approaches to determine the normal ranges of each EAT composition.

## 5 Conclusion

Even if sex-specific individual electrical measurements appear similar in absolute values, the association between inflammation and electrical activity differs significantly between males and females, and this consideration may be relevant for future risk stratification studies of the interaction between EAT and the cardiac environment. Our study provides novel evidence linking the molecular profile of EAT with specific ECG conduction abnormalities. EAT inflammation is strongly associated with delayed late depolarization, particularly in females. These findings highlight inflammation as the main EAT-related factor associated with these electrical disturbances and suggest that ECG parameters may merit further investigation as potential non-invasive correlates of adverse EAT characteristics and treatment response. Together, our results underscore the mutual relationship between EAT remodelling and cardiac electrical activity and emphasize the potential clinical value of integrating ECG assessment with EAT molecular analysis.

## Data Availability

The data the support the findings of this study are available on request from the corresponding author, [SE]. Requests to access these datasets should be directed to Sonia Eiras Penas, sonia.eiras.penas@sergas.es.
