# Supplementary material for: Delayed late ventricular depolarization is associated with inflammation in epicardial Fat Among females: a New avenue in cardiac electrophysiology
Source: Front Cardiovasc Med. 2026 Sep 7;13:1947506. doi: 10.3389/fcvm.2026.1947506 (PMC13590784; doi:10.3389/fcvm.2026.1947506)
Supplement: Supplementary file 1 [file Datasheet1.docx]

Supplementary Material

# 1 Supplementary Data

## 1.1 Supplementary data - Methods

### 1.1.1 Principal Component Analysis

Principal component analysis (PCA) was conducted for the two groups of ECG variables: depolarization and repolarization variables. In the depolarization group, PCA of the 19 ECG-derived depolarization variables identified four principal components with eigenvalues greater than 1, together explaining 68.922% of the total variance. PC1 accounted for 26.901% of the variance, followed by PC2 (21.918%), PC3 (10.953%), and PC4 (9.151%) (Supplementary Table 4). The scree plot demonstrated two clear inflection points: one after the PC2 and the other after the PC4, supporting that PC1-2 are the most important components on the one hand, and the retention of PC3-PC4 for further interpretation on the other hand (Supplementary Figure 1A). However, although six components had eigenvalues slightly above 1, only the first four were retained because they captured the meaningful electrophysiological structure of the dataset. PC5 and PC6 did not contain variables with substantial loadings and therefore lacked biological interpretability.

In a similar way, in the repolarization group, PCA of the 17 repolarization-derived ECG variables identified three principal components collectively explaining 62.119% of the total variance. PC1 accounted for the largest proportion of variance (29.989%), and the scree plot demonstrated a clear inflection after it, followed by PC2 (18.115%) and PC3 (14.015%) (Supplementary Table 4). Another clear inflection in the scree plot was observed after PC5 (Supplementary Figure 2A). However, components after the PC3 lacked coherent electrophysiological structure, and thus were not retained.

#### 1.1.1.1 Component interpretation of the depolarization group

PC1: Late anteroseptal and right-sided depolarization: PC1 was primarily defined by strong positive loadings of S-wave amplitudes and upstroke times in the V2-V3 precordial leads, accompanied by marked negative loadings of R/S amplitude ratios. Contribution analysis showed that S-wave parameters accounted for more than 45% of the variance in PC1. Cos^2^ values indicated excellent representation of S-wave upstroke time in V2 and V3 (Cos^2^ ≈ 0.7). Collectively, these features characterize PC1 as a marker of late anteroseptal depolarization with partial involvement of late right ventricular activation (Table 3, Supplementary Figure 1B).

PC2: Early right ventricular depolarization: PC2 was dominated by strong positive loadings of R variables on V1 and V2. R variables on V1 alone contributed more than 50% of the component’s structure. Additional contributions from R variables on V2 (≈15%) further supported the interpretation of PC2 as reflecting early right ventricular depolarization (Table 3, Supplementary Figure 1C).

PC3: Depolarization heterogeneity: PC3 captured a pattern of depolarization heterogeneity. The strongest negative loadings were observed for QRS right-to-left ratio and the ratio of (the widest QRS in V1 or V2)/(the narrowest QRS in V5 or V6), forming the core of this component, contributing together about 60% of it. QRS duration and QRS dispersion demonstrated moderate loadings and contributed together approximately 20% to the component, suggesting a secondary but meaningful role in capturing variability in depolarization timing. Based on contribution and Cos^2^ metrics, these variables collectively explain its biological relevance as a marker of conduction heterogeneity (Table 3, Supplementary Figure 1D).

PC4: Early anteroseptal depolarization: PC4 was characterized by high positive loadings of R amplitude and R/S amplitude ratio on V3 primarily, supporting the interpretation of PC4 as reflecting early anteroseptal depolarization (Table 3, Supplementary Figure 1E).

#### 1.1.1.2 Component interpretation of the repolarization group

PC1: Regional repolarization ratios: PC1 was dominated by strong positive loadings of the right-to-left ratios of QT, QTc, JT, JTc, Tpe, Tpe/QT, and Tpe/QTc, all exceeding 0.70 (Table 3, Supplementary Figure 2B). These variables contributed more than 90% of the component’s structure. Collectively, PC1 reflects regional repolarization ratios, capturing the shared variance of different repolarization parameters between right and left precordial leads (Table 3, Supplementary Figure 2B).

PC2: Transmural-related repolarization dispersion: PC2 was characterized by high positive loadings of Tpe-related repolarization parameters, showing the strongest contribution (≈ 50%) together. The high loading and contribution of Tpe-related repolarization parameters in PC2 capture the transmural-related repolarization dispersion across precordial leads (Table 3, Supplementary Figure 2C).

PC3: Corrected repolarization durations and dispersions: PC3 was defined by strong loadings of QTc and JTc and their dispersions. Unlike PC2, these variables represent the overall aspect of repolarization phase rather than the timing variability in the repolarization period, especially the lead-dependent variability measured by Tpe-related parameters (Table 3, Supplementary Figure 2D).

#### 1.1.1.3 Individual factor scores and distribution in PCA space - Depolarization group

The distribution of variables across the first two components showed consistent grouping of related ECG features, reflecting the same patterns identified through loadings and contribution analyses (Supplementary Figure 3A). When the population was projected onto the same component space, individuals displayed a wide but organized spread, indicating meaningful variation in multivariate ECG profiles (Supplementary Figure 3B). The combined visualization of variables and individuals further confirmed the coherence of these relationships and demonstrated how the major ECG features contributed to the separation observed within the population.

The distribution of repolarization variables across PC1 and PC2 showed clear clustering of related features (Supplementary Figure 4A). Right-to-left ratios grouped tightly along PC1, whereas absolute values aligned along PC2, confirming the distinct electrophysiological domains captured by each component. Projection of individuals onto the same PCA space revealed a wide but structured spread, indicating meaningful inter-individual variability in repolarization patterns (Supplementary Figure 4B).

# 2 Supplementary Figures and Tables

## 2.1 Supplementary Figures

**Supplementary Table 1.** Sequence 5' --> 3' of primers forward and reverse

| **Primer** | **Sequence 5' --> 3'** |
| --- | --- |
| ACTB forward | TTCTGACCCATGCCCACCAT |
| ACTB reverse | ATGGATGATGATATCGCCGCGCTC |
| ADRB3 forward | TACTCTGCGCTGGCTTTTGA |
| ADRB3 reverse | AAGGCTCAAGCTCACTCCC |
| CD14 forward | GCTGTGTAGGAAAGAAGCTA |
| CD14 reverse | TTTAGAAACGGCTCTAGGTTG |
| CD16 forward | CCTCCTGTCTAGTCGGTTTGG |
| CD16 reverse | TCGAGCACCCTGTACCATTGA |
| CD36 forward | TCTTTCCTGCAGCCCAATG |
| CD36 reverse | AGCCTCTGTTCCAACTGATAGTGA |
| CD68 forward | CTTTGGGCAAGGTTTCTCCTGCC |
| CD68 reverse | CTCCGGATGATGCAGAAAGC |
| COL1A2 forward | TCGCACATGCCGTGACTTG |
| COL1A2 reverse | GATAGCATCCATAGTGCATCCTTG |
| CXCR2 forward | CGAAGGACCGTCTACTCATC |
| CXCR2 reverse | AGTGTGCCCTGAAGAAGAGC |
| DEFA3 forward | TCCCAGAAGTGGTTGTTTCC |
| DEFA3 reverse | CAGAATGCCCAGAGTCTTCC |
| FABP4 forward | TACTGGGCCAGGAATTTGAC |
| FABP4 reverse | GTGGAAGTGACGCCTTTCAT |
| mAChR2 forward | CTCCAGCCATTCTCTTCTGG |
| mAChR2 reverse | GCAACAGGCTCCTTCTTGTC |
| mAChR3 forward | CGCTCCAACAGGAGGAAGTA |
| mAChR3 reverse | GGAGTTGAGGATGGTGCTGT |
| PREF1 forward | CTGGACGGTGGCCTCTATGAATG |
| PREF1 reverse | ATCATCCACGCAGGTGCCTC |

ACTB: b-actin; ADRB3: B3 adrenergic receptor; COL1A2: collagen type I alpha 2 chain; CXCR2: CXC chemokine receptor 2; DEFA3: defensin alpha 3; FABP4: fatty acid binding protein 4; mAChR2: M2 muscarinic acetylcholine receptor; mAChR3: M3 muscarinic acetylcholine receptor; PREF1: preadipocyte factor-1.

**Supplementary Table 2.** descriptive analysis of all continuous variables in the total population:

| **Variable** | **n** | **Mean** | **SD** | **Median** | **Q1** | **Q3** |
| --- | --- | --- | --- | --- | --- | --- |
| Age (years) | 95 | 67.874 | 8.869 | 71 | 63 | 74 |
| BMI (Kg/m^2^) | 95 | 28.644 | 3.971 | 29.032 | 24.987 | 31.840 |
| R1_A (mm) | 73 | 2.030 | 2.074 | 1.330 | 0.750 | 2.500 |
| S1_A (mm) | 93 | 9.427 | 5.048 | 8.500 | 6.000 | 12.500 |
| R/S_1 (mm) | 72 | 0.386 | 0.814 | 0.180 | 0.079 | 0.266 |
| R1_PT (sec) | 73 | 0.029 | 0.021 | 0.020 | 0.020 | 0.030 |
| S1_UT (sec) | 86 | 0.049 | 0.017 | 0.050 | 0.040 | 0.058 |
| R2_A (mm) | 83 | 4.840 | 3.940 | 3.920 | 1.790 | 6.773 |
| S2_A (mm) | 92 | 11.349 | 7.467 | 9.169 | 6.585 | 14.375 |
| R/S_2 (mm) | 82 | 0.736 | 0.889 | 0.403 | 0.184 | 0.857 |
| R2_PT (sec) | 83 | 0.028 | 0.010 | 0.027 | 0.020 | 0.035 |
| S2_UT (sec) | 89 | 0.046 | 0.019 | 0.040 | 0.037 | 0.050 |
| R3_A (mm) | 89 | 8.971 | 6.942 | 7.170 | 3.500 | 13.500 |
| S3_A (mm) | 93 | 12.178 | 8.718 | 9.330 | 6.250 | 15.750 |
| R/S_3 (mm) | 87 | 1.418 | 1.844 | 0.663 | 0.269 | 1.852 |
| R3_PT (sec) | 89 | 0.032 | 0.011 | 0.030 | 0.020 | 0.040 |
| S3_UT (sec) | 93 | 0.044 | 0.017 | 0.040 | 0.030 | 0.050 |
| QRS_R/L (sec) | 95 | 1.046 | 0.141 | 1.058 | 0.966 | 1.124 |
| WQRS/NQRS (sec) | 95 | 1.194 | 0.219 | 1.194 | 1.043 | 1.297 |
| QRS (sec) | 95 | 0.103 | 0.021 | 0.100 | 0.091 | 0.111 |
| QRSd (sec) | 95 | 0.029 | 0.014 | 0.028 | 0.020 | 0.036 |
| QT_R/L (sec) | 95 | 0.971 | 0.142 | 0.976 | 0.941 | 1.021 |
| QTc_R/L (sec) | 95 | 0.975 | 0.147 | 0.980 | 0.938 | 1.025 |
| JT_R/L (sec) | 95 | 0.941 | 0.164 | 0.958 | 0.869 | 1.029 |
| JTc_R/L (sec) | 95 | 0.953 | 0.172 | 0.973 | 0.888 | 1.030 |
| Tpe_R/L (sec) | 95 | 1.074 | 0.309 | 1.065 | 0.895 | 1.224 |
| Tpe/QT_R/L (sec) | 95 | 1.083 | 0.295 | 1.070 | 0.893 | 1.229 |
| Tpe/QTc_R/L (sec) | 95 | 1.083 | 0.307 | 1.044 | 0.887 | 1.231 |
| QT (sec) | 95 | 0.385 | 0.036 | 0.380 | 0.357 | 0.408 |
| QTc (sec) | 95 | 0.421 | 0.029 | 0.419 | 0.400 | 0.441 |
| JT (sec) | 95 | 0.281 | 0.032 | 0.281 | 0.256 | 0.303 |
| JTc (sec) | 95 | 0.317 | 0.032 | 0.318 | 0.297 | 0.342 |
| Tpe (sec) | 95 | 0.072 | 0.011 | 0.071 | 0.065 | 0.077 |
| Tpe/QT (sec) | 95 | 0.186 | 0.026 | 0.188 | 0.166 | 0.203 |
| Tpe/QTc (sec) | 95 | 0.171 | 0.027 | 0.169 | 0.155 | 0.188 |
| QTcd (sec) | 95 | 0.066 | 0.039 | 0.061 | 0.040 | 0.086 |
| JTcd (sec) | 95 | 0.070 | 0.044 | 0.060 | 0.043 | 0.086 |
| dTpe (sec) | 95 | 0.034 | 0.018 | 0.030 | 0.020 | 0.040 |
| NTproBNP (pg/ml) | 76 | 1239.355 | 1889.240 | 590.000 | 257.750 | 1245.000 |
| HbA1C% | 71 | 6.080 | 1.026 | 5.800 | 5.550 | 6.250 |
| LVEF% | 82 | 56.805 | 10.786 | 59.000 | 50.500 | 63.000 |

BMI: body mass index; dTpe: dispersion of T peak - T end; HbA1C: Haemoglobin A1C; JTc: corrected JT; JTcd: dispersion of corrected JT; JT R/L: JT right (V1+V2+V3)-to-left (V4+V5+V6) ratio; JTc R/L: corrected JT right (V1+V2+V3)-to-left (V4+V5+V6) ratio; LVEF: left ventricular ejection fraction; NTproBNP: N‑Terminal pro–B‑type Natriuretic Peptide; Q1: first quartile; Q3: third quartile; QRSd: QRS dispersion; QRS R/L: QRS right (V1+V2+V3)-to-left (V4+V5+V6) ratio; QTc: corrected QT; QTcd: dispersion of corrected QT; QT R/L: QT right (V1+V2+V3)-to-left (V4+V5+V6) ratio; QTc R/L: corrected QT right (V1+V2+V3)-to-left (V4+V5+V6) ratio; R1 A: R-wave amplitude in V1; R2 A: R-wave amplitude in V2; R3 A: R-wave amplitude in V3; R1 PT: R-wave peak time in V1; R2 PT: R-wave peak time in V2; R3 PT: R-wave peak time in V3; R/S 1: R/S amplitude ratio in V1; R/S 2: R/S amplitude ratio in V2; R/S 3: R/S amplitude ratio in V3; SD: standard deviation; S1 A: S-wave amplitude in V1; S2 A: S-wave amplitude in V2; S3 A: S-wave amplitude in V3; S1 UT: S-wave upstroke time in V1; S2 UT: S-wave upstroke time in V2; S3 UT: S-wave upstroke time in V3; Tpe: T peak - T end; Tpe/QT R/L: Tpe/QT right (V1+V2+V3)-to-left (V4+V5+V6) ratio; Tpe/QTc R/L: Tpe/QTc right (V1+V2+V3)-to-left (V4+V5+V6) ratio; Tpe R/L: Tpe right (V1+V2+V3)-to-left (V4+V5+V6) ratio; WQRS/NQRS: the widest QRS (in V1 or V2)/the narrowest QRS (in V5 or V6).

**Supplementary Table 3.** sex-stratified descriptive analysis of all continuous variables, and sex differences assessed using the Mann-Whitney U test:

| **Sex** | **Variable** | **Mean** | **SD** | **Median** | **Q1** | **Q3** | **Min** | **Max** | **U_statistic** | **p_value** |
| --- | --- | --- | --- | --- | --- | --- | --- | --- | --- | --- |
| Males | Age (years) | 66.169 | 9.149 | 66 | 61 | 73 | 38 | 84 | 658.000 | 0.011 |
| Females | Age (years) | 71.567 | 7.045 | 72 | 69 | 77 | 56 | 85 | 658.000 | 0.011 |
| Males | BMI (Kg/m^2^) | 29.172 | 3.674 | 29.385 | 26.235 | 32.298 | 21.336 | 40.083 | 1211.000 | 0.059 |
| Females | BMI (Kg/m^2^) | 27.501 | 4.398 | 27.127 | 23.997 | 31.239 | 20.546 | 38.055 | 1211.000 | 0.059 |
| Males | R1_A (mm) | 2.193 | 2.307 | 1.250 | 0.770 | 2.969 | 0.250 | 12.920 | 626.000 | 0.548 |
| Females | R1_A (mm) | 1.674 | 1.426 | 1.330 | 0.625 | 1.835 | 0.500 | 6.250 | 626.000 | 0.548 |
| Males | S1_A (mm) | 9.029 | 5.271 | 8.250 | 4.875 | 12.500 | 1.000 | 24.330 | 774.500 | 0.162 |
| Females | S1_A (mm) | 10.264 | 4.513 | 9.085 | 7.373 | 13.128 | 2.170 | 20.500 | 774.500 | 0.162 |
| Males | R/S_1 (mm) | 0.465 | 0.962 | 0.184 | 0.096 | 0.267 | 0.015 | 5.742 | 643.000 | 0.340 |
| Females | R/S_1 (mm) | 0.217 | 0.275 | 0.176 | 0.073 | 0.238 | 0.028 | 1.375 | 643.000 | 0.340 |
| Males | R1_PT (sec) | 0.032 | 0.024 | 0.024 | 0.020 | 0.030 | 0.007 | 0.100 | 736.000 | 0.052 |
| Females | R1_PT (sec) | 0.023 | 0.014 | 0.020 | 0.017 | 0.024 | 0.010 | 0.080 | 736.000 | 0.052 |
| Males | S1_UT (sec) | 0.049 | 0.017 | 0.050 | 0.040 | 0.060 | 0.013 | 0.105 | 873.500 | 0.571 |
| Females | S1_UT (sec) | 0.048 | 0.017 | 0.044 | 0.040 | 0.051 | 0.023 | 0.090 | 873.500 | 0.571 |
| Males | R2_A (mm) | 5.203 | 4.074 | 4.375 | 2.000 | 6.875 | 0.583 | 20.170 | 885.500 | 0.157 |
| Females | R2_A (mm) | 4.043 | 3.575 | 2.750 | 1.500 | 5.540 | 0.375 | 13.000 | 885.500 | 0.157 |
| Males | S2_A (mm) | 11.867 | 7.998 | 9.438 | 6.270 | 16.855 | 1.000 | 36.580 | 1003.500 | 0.543 |
| Females | S2_A (mm) | 10.278 | 6.217 | 9.000 | 6.940 | 11.651 | 1.500 | 31.250 | 1003.500 | 0.543 |
| Males | R/S_2 (mm) | 0.768 | 0.861 | 0.443 | 0.208 | 0.954 | 0.036 | 3.571 | 815.500 | 0.386 |
| Females | R/S_2 (mm) | 0.667 | 0.961 | 0.351 | 0.175 | 0.741 | 0.026 | 3.887 | 815.500 | 0.386 |
| Males | R2_PT (sec) | 0.029 | 0.011 | 0.030 | 0.020 | 0.037 | 0.010 | 0.060 | 869.000 | 0.201 |
| Females | R2_PT (sec) | 0.026 | 0.008 | 0.023 | 0.020 | 0.030 | 0.013 | 0.043 | 869.000 | 0.201 |
| Males | S2_UT (sec) | 0.047 | 0.020 | 0.040 | 0.037 | 0.059 | 0.015 | 0.120 | 912.500 | 0.813 |
| Females | S2_UT (sec) | 0.044 | 0.015 | 0.040 | 0.036 | 0.050 | 0.020 | 0.093 | 912.500 | 0.813 |
| Males | R3_A (mm) | 9.678 | 7.475 | 7.920 | 3.500 | 14.625 | 0.330 | 43.670 | 998.000 | 0.205 |
| Females | R3_A (mm) | 7.430 | 5.414 | 5.625 | 3.105 | 10.625 | 0.670 | 18.500 | 998.000 | 0.205 |
| Males | S3_A (mm) | 12.487 | 8.787 | 8.563 | 6.250 | 16.313 | 2.000 | 36.500 | 968.000 | 0.743 |
| Females | S3_A (mm) | 11.497 | 8.678 | 10.250 | 7.170 | 13.330 | 2.170 | 41.580 | 968.000 | 0.743 |
| Males | R/S_3 (mm) | 1.443 | 1.848 | 0.726 | 0.315 | 1.872 | 0.040 | 9.750 | 899.000 | 0.417 |
| Females | R/S_3 (mm) | 1.364 | 1.871 | 0.606 | 0.191 | 1.651 | 0.062 | 6.949 | 899.000 | 0.417 |
| Males | R3_PT (sec) | 0.033 | 0.011 | 0.033 | 0.023 | 0.040 | 0.013 | 0.070 | 1052.500 | 0.076 |
| Females | R3_PT (sec) | 0.029 | 0.011 | 0.030 | 0.020 | 0.038 | 0.010 | 0.060 | 1052.500 | 0.076 |
| Males | S3_UT (sec) | 0.045 | 0.018 | 0.040 | 0.032 | 0.052 | 0.010 | 0.110 | 1029.000 | 0.400 |
| Females | S3_UT (sec) | 0.042 | 0.017 | 0.040 | 0.030 | 0.050 | 0.020 | 0.093 | 1029.000 | 0.400 |
| Males | QRS_R/L (sec) | 1.043 | 0.159 | 1.042 | 0.960 | 1.123 | 0.571 | 1.533 | 934.000 | 0.746 |
| Females | QRS_R/L (sec) | 1.053 | 0.092 | 1.058 | 0.969 | 1.123 | 0.859 | 1.271 | 934.000 | 0.746 |
| Males | WQRS/NQRS (sec) | 1.199 | 0.245 | 1.194 | 1.000 | 1.333 | 0.826 | 2.192 | 955.500 | 0.879 |
| Females | WQRS/NQRS (sec) | 1.185 | 0.150 | 1.193 | 1.070 | 1.283 | 0.880 | 1.429 | 955.500 | 0.879 |
| Males | QRS (sec) | 0.106 | 0.021 | 0.102 | 0.095 | 0.116 | 0.074 | 0.181 | 1247.500 | 0.029 |
| Females | QRS (sec) | 0.098 | 0.019 | 0.095 | 0.087 | 0.102 | 0.068 | 0.161 | 1247.500 | 0.029 |
| Males | QRSd (sec) | 0.031 | 0.015 | 0.030 | 0.020 | 0.040 | 0.007 | 0.087 | 1166.500 | 0.125 |
| Females | QRSd (sec) | 0.026 | 0.009 | 0.024 | 0.020 | 0.030 | 0.012 | 0.050 | 1166.500 | 0.125 |
| Males | QT_R/L (sec) | 0.973 | 0.151 | 0.983 | 0.944 | 1.034 | 0.367 | 1.642 | 1130.500 | 0.215 |
| Females | QT_R/L (sec) | 0.967 | 0.123 | 0.965 | 0.934 | 0.994 | 0.665 | 1.426 | 1130.500 | 0.215 |
| Males | QTc_R/L (sec) | 0.980 | 0.160 | 0.985 | 0.939 | 1.032 | 0.367 | 1.693 | 1084.000 | 0.385 |
| Females | QTc_R/L (sec) | 0.963 | 0.115 | 0.970 | 0.935 | 1.000 | 0.671 | 1.206 | 1084.000 | 0.385 |
| Males | JT_R/L (sec) | 0.944 | 0.185 | 0.967 | 0.876 | 1.036 | 0.331 | 1.608 | 1067.000 | 0.464 |
| Females | JT_R/L (sec) | 0.936 | 0.106 | 0.940 | 0.862 | 0.989 | 0.623 | 1.167 | 1067.000 | 0.464 |
| Males | JTc_R/L (sec) | 0.961 | 0.190 | 0.973 | 0.884 | 1.059 | 0.338 | 1.729 | 1106.000 | 0.296 |
| Females | JTc_R/L (sec) | 0.936 | 0.126 | 0.967 | 0.901 | 0.991 | 0.634 | 1.260 | 1106.000 | 0.296 |
| Males | Tpe_R/L (sec) | 1.092 | 0.325 | 1.083 | 0.917 | 1.237 | 0.327 | 2.500 | 1076.000 | 0.421 |
| Females | Tpe_R/L (sec) | 1.034 | 0.271 | 1.043 | 0.866 | 1.197 | 0.542 | 1.667 | 1076.000 | 0.421 |
| Males | Tpe/QT_R/L (sec) | 1.094 | 0.306 | 1.081 | 0.893 | 1.229 | 0.296 | 2.293 | 1037.000 | 0.622 |
| Females | Tpe/QT_R/L (sec) | 1.059 | 0.274 | 1.055 | 0.880 | 1.221 | 0.552 | 1.682 | 1037.000 | 0.622 |
| Males | Tpe/QTc_R/L (sec) | 1.089 | 0.309 | 1.044 | 0.886 | 1.230 | 0.296 | 2.224 | 1009.000 | 0.789 |
| Females | Tpe/QTc_R/L (sec) | 1.070 | 0.307 | 1.050 | 0.896 | 1.230 | 0.550 | 2.053 | 1009.000 | 0.789 |
| Males | QT (sec) | 0.386 | 0.037 | 0.380 | 0.355 | 0.412 | 0.304 | 0.482 | 1020.000 | 0.722 |
| Females | QT (sec) | 0.383 | 0.036 | 0.378 | 0.358 | 0.402 | 0.324 | 0.498 | 1020.000 | 0.722 |
| Males | QTc (sec) | 0.418 | 0.027 | 0.417 | 0.399 | 0.435 | 0.357 | 0.498 | 804.000 | 0.172 |
| Females | QTc (sec) | 0.427 | 0.033 | 0.426 | 0.407 | 0.448 | 0.360 | 0.503 | 804.000 | 0.172 |
| Males | JT (sec) | 0.279 | 0.033 | 0.278 | 0.254 | 0.299 | 0.213 | 0.351 | 849.000 | 0.315 |
| Females | JT (sec) | 0.285 | 0.031 | 0.284 | 0.263 | 0.307 | 0.222 | 0.340 | 849.000 | 0.315 |
| Males | JTc (sec) | 0.311 | 0.032 | 0.310 | 0.289 | 0.333 | 0.224 | 0.381 | 644.500 | 0.008 |
| Females | JTc (sec) | 0.329 | 0.028 | 0.329 | 0.309 | 0.350 | 0.263 | 0.376 | 644.500 | 0.008 |
| Males | Tpe (sec) | 0.072 | 0.011 | 0.071 | 0.066 | 0.078 | 0.052 | 0.100 | 1085.500 | 0.378 |
| Females | Tpe (sec) | 0.070 | 0.012 | 0.069 | 0.061 | 0.076 | 0.046 | 0.097 | 1085.500 | 0.378 |
| Males | Tpe/QT (sec) | 0.188 | 0.025 | 0.189 | 0.168 | 0.201 | 0.131 | 0.239 | 1035.000 | 0.634 |
| Females | Tpe/QT (sec) | 0.184 | 0.028 | 0.188 | 0.163 | 0.203 | 0.137 | 0.241 | 1035.000 | 0.634 |
| Males | Tpe/QTc (sec) | 0.173 | 0.026 | 0.171 | 0.156 | 0.192 | 0.126 | 0.231 | 1139.000 | 0.191 |
| Females | Tpe/QTc (sec) | 0.165 | 0.029 | 0.164 | 0.143 | 0.177 | 0.111 | 0.235 | 1139.000 | 0.191 |
| Males | QTcd (sec) | 0.065 | 0.033 | 0.061 | 0.041 | 0.084 | 0.014 | 0.155 | 1007.500 | 0.798 |
| Females | QTcd (sec) | 0.069 | 0.049 | 0.060 | 0.034 | 0.085 | 0.010 | 0.234 | 1007.500 | 0.798 |
| Males | JTcd (sec) | 0.069 | 0.042 | 0.065 | 0.046 | 0.088 | 0.009 | 0.300 | 992.500 | 0.892 |
| Females | JTcd (sec) | 0.073 | 0.049 | 0.055 | 0.040 | 0.084 | 0.016 | 0.240 | 992.500 | 0.892 |
| Males | dTpe (sec) | 0.033 | 0.016 | 0.030 | 0.020 | 0.040 | 0.010 | 0.080 | 928.000 | 0.709 |
| Females | dTpe (sec) | 0.036 | 0.021 | 0.030 | 0.020 | 0.040 | 0.010 | 0.110 | 928.000 | 0.709 |
| Males | NTproBNP (pg/ml) | 1153.673 | 1897.561 | 503.500 | 165.000 | 1082.250 | 19.000 | 11585.000 | 483.000 | 0.116 |
| Females | NTproBNP (pg/ml) | 1425.000 | 1897.889 | 706.500 | 504.750 | 1314.000 | 199.000 | 7176.000 | 483.000 | 0.116 |
| Males | HbA1C% | 6.259 | 1.173 | 5.900 | 5.700 | 6.700 | 4.800 | 11.500 | 717.500 | 0.026 |
| Females | HbA1C% | 5.682 | 0.350 | 5.700 | 5.425 | 6.000 | 5.000 | 6.200 | 717.500 | 0.026 |
| Males | LVEF% | 54.842 | 10.858 | 58.000 | 50.000 | 63.000 | 24.000 | 67.000 | 471.500 | 0.015 |
| Females | LVEF% | 61.280 | 9.356 | 62.000 | 56.000 | 67.000 | 39.000 | 77.000 | 471.500 | 0.015 |

BMI: body mass index; dTpe: dispersion of T peak - T end; HbA1C: Haemoglobin A1C; JTc: corrected JT; JTcd: dispersion of corrected JT; JT R/L: JT right (V1+V2+V3)-to-left (V4+V5+V6) ratio; JTc R/L: corrected JT right (V1+V2+V3)-to-left (V4+V5+V6) ratio; LVEF: left ventricular ejection fraction; Max: maximum; Min: minimum; NTproBNP: N‑Terminal pro–B‑type Natriuretic Peptide; Q1: first quartile; Q3: third quartile; QRSd: QRS dispersion; QRS R/L: QRS right (V1+V2+V3)-to-left (V4+V5+V6) ratio; QTc: corrected QT; QTcd: dispersion of corrected QT; QT R/L: QT right (V1+V2+V3)-to-left (V4+V5+V6) ratio; QTc R/L: corrected QT right (V1+V2+V3)-to-left (V4+V5+V6) ratio; R1 A: R-wave amplitude in V1; R2 A: R-wave amplitude in V2; R3 A: R-wave amplitude in V3; R1 PT: R-wave peak time in V1; R2 PT: R-wave peak time in V2; R3 PT: R-wave peak time in V3; R/S 1: R/S amplitude ratio in V1; R/S 2: R/S amplitude ratio in V2; R/S 3: R/S amplitude ratio in V3; SD: standard deviation; S1 A: S-wave amplitude in V1; S2 A: S-wave amplitude in V2; S3 A: S-wave amplitude in V3; S1 UT: S-wave upstroke time in V1; S2 UT: S-wave upstroke time in V2; S3 UT: S-wave upstroke time in V3; Tpe: T peak - T end; Tpe/QT R/L: Tpe/QT right (V1+V2+V3)-to-left (V4+V5+V6) ratio; Tpe/QTc R/L: Tpe/QTc right (V1+V2+V3)-to-left (V4+V5+V6) ratio; Tpe R/L: Tpe right (V1+V2+V3)-to-left (V4+V5+V6) ratio; WQRS/NQRS: the widest QRS (in V1 or V2)/the narrowest QRS (in V5 or V6).

**Supplementary Table 4.** Eigenvalues and explained variance of the principal components

| **Components (Dimensions)** | **Eigenvalue** | **Percentage of variance %** | **Cumulative percentage of variance %** |
| --- | --- | --- | --- |
| **Depolarization variables** | | | |
| PC1 (Dim 1) | 5.111 | 26.901 | 26.901 |
| PC2 (Dim 2) | 4.164 | 21.918 | 48.818 |
| PC3 (Dim 3) | 2.081 | 10.953 | 59.771 |
| PC4 (Dim 4) | 1.739 | 9.151 | 68.922 |
| PC5 (Dim 5) | 1.116 | 5.875 | 74.797 |
| PC6 (Dim 6) | 1.013 | 5.331 | 80.128 |
| **Repolarization variables** | | | |
| PC1 (Dim 1) | 5.098 | 29.989 | 29.989 |
| PC2 (Dim 2) | 3.080 | 18.115 | 48.104 |
| PC3 (Dim 3) | 2.383 | 14.015 | 62.119 |
| PC4 (Dim 4) | 1.918 | 11.280 | 73.399 |
| PC5 (Dim 5) | 1.262 | 7.422 | 80.821 |
| PC6 (Dim 6) | 1.076 | 6.328 | 87.149 |

Dim: dimension; PC: principal component.

**Supplementary Table 5.** Spearman correlation analyses between the PCA components and EAT markers:

|  | **Population** | **PCA component** | **EAT Marker** | **r** | **p** | **Adj.p** |
| --- | --- | --- | --- | --- | --- | --- |
| Depolarization | All | Depolarization - PC1 | FABP4 | -0.256 | 0.012 | 0.524 |
|  | All | Depolarization - PC1 | CD36 | -0.230 | 0.025 | 0.524 |
|  | All | Depolarization - PC1 | CD14 | 0.219 | 0.033 | 0.524 |
|  | All | Depolarization - PC1 | CD16 | 0.210 | 0.046 | 0.526 |
|  | Female | Depolarization - PC1 | CD16 | 0.533 | 0.003 | 0.068 |
|  | Female | Depolarization - PC1 | DEFA3 | 0.520 | 0.004 | 0.068 |
|  | Female | Depolarization - PC1 | mACHR3 | 0.498 | 0.006 | 0.068 |
|  | Female | Depolarization - PC1 | COL1A2 | 0.498 | 0.006 | 0.068 |
|  | Female | Depolarization - PC1 | CD14 | 0.444 | 0.015 | 0.126 |
|  | Female | Depolarization - PC1 | mACHR2 | 0.439 | 0.016 | 0.126 |
|  | Female | Depolarization - PC1 | ADRB3 | 0.430 | 0.018 | 0.126 |
|  | Female | Depolarization - PC1 | CXCR2 | 0.395 | 0.035 | 0.209 |
|  | Male | Depolarization - PC2 | CD36 | 0.254 | 0.042 | 0.924 |
| Repolarization | All | Repolarization - PC2 | PREF1 | -0.251 | 0.017 | 0.425 |
|  | All | Repolarization - PC1 | CXCR2 | 0.247 | 0.019 | 0.425 |
|  | All | Repolarization - PC1 | DEFA3 | 0.211 | 0.041 | 0.431 |
|  | Female | Repolarization - PC2 | PREF1 | -0.492 | 0.006 | 0.301 |
|  | Female | Repolarization - PC1 | CXCR2 | 0.448 | 0.016 | 0.373 |
|  | Female | Repolarization - PC1 | CD36 | -0.402 | 0.028 | 0.456 |
|  | Female | Repolarization - PC1 | mACHR2 | 0.377 | 0.041 | 0.469 |
|  | Female | Repolarization - PC1 | ADRB3 | 0.364 | 0.049 | 0.469 |

ADRB3: B3 adrenergic receptor; COL1A2: collagen type I alpha 2 chain; CXCR2: CXC chemokine receptor 2; DEFA3: defensin alpha 3; FABP4: fatty acid binding protein 4; mAChR2: M2 muscarinic acetylcholine receptor; mAChR3: M3 muscarinic acetylcholine receptor; PREF1: preadipocyte factor-1.

**Supplementary Table 6.** Spearman correlation analyses between the ECG repolarization variables and EAT molecular profile:

| **Population** | **ECG variable** | **EAT Marker** | **r** | **p** | **Adj.p** |
| --- | --- | --- | --- | --- | --- |
| All | Tpe/QT | COL1A2 | -0.294 | 0.004 | 0.426 |
| All | JTc | FABP4 | 0.266 | 0.009 | 0.426 |
| All | QTc_R/L | CD14 | 0.243 | 0.018 | 0.426 |
| All | JTcd | CD68 | 0.242 | 0.018 | 0.426 |
| All | QTcd | CD68 | 0.240 | 0.019 | 0.426 |
| All | JT_R/L | CD36 | -0.235 | 0.022 | 0.426 |
| All | JTc_R/L | CD36 | -0.233 | 0.024 | 0.426 |
| All | QTcd | ADRB3 | 0.236 | 0.024 | 0.426 |
| All | QTc_R/L | CD16 | 0.228 | 0.030 | 0.426 |
| All | QTcd | mACHR3 | 0.223 | 0.031 | 0.426 |
| All | QTcd | DEFA3 | 0.222 | 0.032 | 0.426 |
| All | Tpe/QTc_R/L | PREF1 | -0.222 | 0.035 | 0.426 |
| All | QTcd | mACHR2 | 0.218 | 0.037 | 0.426 |
| All | QTc_R/L | CXCR2 | 0.220 | 0.038 | 0.426 |
| All | Tpe/QTc_R/L | CD36 | -0.213 | 0.038 | 0.426 |
| All | Tpe/QT_R/L | CD36 | -0.211 | 0.040 | 0.426 |
| All | Tpe/QT_R/L | PREF1 | -0.213 | 0.043 | 0.426 |
| All | QTcd | CXCR2 | 0.211 | 0.046 | 0.426 |
| All | QTc_R/L | DEFA3 | 0.206 | 0.047 | 0.426 |
| All | QTc_R/L | ADRB3 | 0.208 | 0.048 | 0.426 |
| Female | JT | mACHR2 | -0.379 | 0.040 | 0.557 |
| Female | JTc | FABP4 | 0.465 | 0.010 | 0.446 |
| Female | JTc | CD36 | 0.464 | 0.011 | 0.446 |
| Female | QT_R/L | CD14 | 0.383 | 0.037 | 0.557 |
| Female | QT_R/L | DEFA3 | 0.382 | 0.042 | 0.557 |
| Female | QTc_R/L | CXCR2 | 0.425 | 0.022 | 0.557 |
| Female | QTc_R/L | DEFA3 | 0.420 | 0.024 | 0.557 |
| Female | QTc_R/L | ADRB3 | 0.392 | 0.033 | 0.557 |
| Female | QTc_R/L | mACHR2 | 0.390 | 0.034 | 0.557 |
| Female | Tpe | PREF1 | -0.392 | 0.032 | 0.557 |
| Female | Tpe/QT_R/L | CD36 | -0.442 | 0.015 | 0.514 |
| Female | Tpe/QTc | PREF1 | -0.462 | 0.011 | 0.446 |
| Female | Tpe/QTc_R/L | CD36 | -0.476 | 0.008 | 0.446 |
| Female | Tpe/QTc_R/L | PREF1 | -0.366 | 0.047 | 0.557 |
| Female | Tpe_R/L | CD36 | -0.487 | 0.007 | 0.446 |
| Female | Tpe_R/L | PREF1 | -0.366 | 0.048 | 0.557 |
| Male | JTcd | DEFA3 | 0.262 | 0.035 | 0.829 |
| Male | JTcd | CD68 | 0.261 | 0.035 | 0.829 |
| Male | JTcd | PREF1 | 0.267 | 0.038 | 0.829 |
| Male | QTc_R/L | PREF1 | 0.257 | 0.046 | 0.829 |
| Male | QTcd | PREF1 | 0.314 | 0.014 | 0.829 |
| Male | QTcd | DEFA3 | 0.295 | 0.017 | 0.829 |
| Male | QTcd | ADRB3 | 0.267 | 0.038 | 0.829 |
| Male | Tpe/QT | COL1A2 | -0.366 | 0.003 | 0.560 |

ADRB3: B3 adrenergic receptor; COL1A2: collagen type I alpha 2 chain; CXCR2: CXC chemokine receptor 2; DEFA3: defensin alpha 3; dTpe: dispersion of T peak - T end; FABP4: fatty acid binding protein 4; JTc: corrected JT; JTcd: dispersion of corrected JT; JT R/L: JT right (V1+V2+V3)-to-left (V4+V5+V6) ratio; JTc R/L: corrected JT right (V1+V2+V3)-to-left (V4+V5+V6) ratio; mAChR2: M2 muscarinic acetylcholine receptor; mAChR3: M3 muscarinic acetylcholine receptor; PREF1: preadipocyte factor-1; QTc: corrected QT; QTcd: dispersion of corrected QT; QT R/L: QT right (V1+V2+V3)-to-left (V4+V5+V6) ratio; QTc R/L: corrected QT right (V1+V2+V3)-to-left (V4+V5+V6) ratio; Tpe: T peak - T end; Tpe/QT R/L: Tpe/QT right (V1+V2+V3)-to-left (V4+V5+V6) ratio; Tpe/QTc R/L: Tpe/QTc right (V1+V2+V3)-to-left (V4+V5+V6) ratio; Tpe R/L: Tpe right (V1+V2+V3)-to-left (V4+V5+V6) ratio.

**Supplementary Table 7.** Sex-specific regression slopes and interaction effects for ECG-EAT associations

| **ECG** | **Marker** | **intf_beta** | **intf_ci_low** | **intf_ci_high** | **intf_p** | **intm_beta** | **intm_ci_low** | **intm_ci_high** | **intm_p** | **f_beta** | **f_ci_low** | **f_ci_high** | **f_p** | **m_beta** | **m_ci_low** | **m_ci_high** | **m_p** |
| --- | --- | --- | --- | --- | --- | --- | --- | --- | --- | --- | --- | --- | --- | --- | --- | --- | --- |
| S2_UT | CD14 | 0.867 | 0.151 | 1.583 | 0.018 | 0.564 | -0.026 | 1.153 | 0.061 | 1.881 | 0.722 | 3.040 | 0.002 | 0.264 | -0.419 | 0.947 | 0.443 |
| S2_UT | CD16 | 1.498 | 0.294 | 2.702 | 0.015 | 1.105 | 0.077 | 2.132 | 0.035 | 3.396 | 1.492 | 5.299 | 0.001 | 0.516 | -0.677 | 1.709 | 0.390 |
| S2_UT | COL1A2 | 0.790 | 0.170 | 1.410 | 0.013 | 0.533 | 0.023 | 1.043 | 0.041 | 1.255 | 0.261 | 2.248 | 0.015 | 0.396 | -0.211 | 1.003 | 0.197 |
| S2_UT | DEFA3 | 2.232 | 0.258 | 4.206 | 0.027 | 1.714 | 0.084 | 3.344 | 0.040 | 5.088 | 2.116 | 8.060 | 0.002 | 0.894 | -1.038 | 2.826 | 0.358 |
| S2_UT | mACHR3 | 1.459 | -0.174 | 3.092 | 0.079 | 1.268 | -0.073 | 2.609 | 0.063 | 3.992 | 1.577 | 6.407 | 0.002 | 0.505 | -1.089 | 2.100 | 0.528 |
| S3_UT | CD14 | 0.894 | 0.162 | 1.627 | 0.017 | 0.488 | -0.134 | 1.110 | 0.123 | 1.835 | 0.811 | 2.858 | 0.001 | 0.062 | -0.693 | 0.817 | 0.870 |
| S3_UT | CD16 | 1.523 | 0.260 | 2.786 | 0.019 | 0.903 | -0.199 | 2.004 | 0.107 | 3.390 | 1.799 | 4.982 | 0.000 | -0.041 | -1.406 | 1.324 | 0.952 |
| S3_UT | COL1A2 | 0.627 | 0.004 | 1.251 | 0.049 | 0.377 | -0.152 | 0.907 | 0.160 | 1.192 | 0.288 | 2.096 | 0.012 | 0.122 | -0.524 | 0.768 | 0.708 |
| S3_UT | DEFA3 | 1.920 | -0.129 | 3.969 | 0.066 | 1.081 | -0.658 | 2.821 | 0.220 | 4.886 | 2.338 | 7.433 | 0.001 | -0.259 | -2.411 | 1.892 | 0.810 |
| S3_UT | mACHR3 | 1.586 | -0.092 | 3.264 | 0.064 | 1.176 | -0.246 | 2.597 | 0.104 | 4.131 | 2.089 | 6.173 | 0.000 | 0.002 | -1.769 | 1.772 | 0.999 |

ci: 95% confidence interval; COL1A2: collagen type I alpha 2 chain; DEFA3: defensin alpha 3; f_beta: female slope from sex-stratified models; intf_beta: female sex-interaction term from a combined sex-interaction model; intm_beta: male sex-interaction term from a combined sex-interaction model; mAChR3: M3 muscarinic acetylcholine receptor; m_beta: male slope from sex-stratified models; S2 UT: S-wave upstroke time in V2; S3 UT: S-wave upstroke time in V3.

**Supplementary Table 8.** Univariable covariate models assessing the effect of individual clinical factors on the ECG × Sex interaction

| **A: Female population** | | | | | | |
| --- | --- | --- | --- | --- | --- | --- |
| **Marker** | **ECG** | **clinical factor** | **beta** | **ci_low** | **ci_high** | **p_value** |
| CD14 | S2_UT | CAD | 0.938 | 0.255 | 1.621 | 0.008 |
| CD14 | S2_UT | HTN | 0.908 | 0.195 | 1.622 | 0.013 |
| CD14 | S2_UT | HFpEF | 0.903 | 0.188 | 1.619 | 0.014 |
| CD14 | S2_UT | VHD | 0.895 | 0.185 | 1.605 | 0.014 |
| CD14 | S2_UT | AF | 0.890 | 0.164 | 1.617 | 0.017 |
| CD14 | S2_UT | DLP | 0.870 | 0.146 | 1.593 | 0.019 |
| CD14 | S2_UT | Age | 0.868 | 0.136 | 1.600 | 0.021 |
| CD14 | S2_UT | BMI | 0.819 | 0.096 | 1.541 | 0.027 |
| CD14 | S2_UT | DM | 0.799 | 0.055 | 1.543 | 0.036 |
| CD16 | S2_UT | CAD | 1.579 | 0.405 | 2.753 | 0.009 |
| CD16 | S2_UT | HTN | 1.553 | 0.350 | 2.756 | 0.012 |
| CD16 | S2_UT | VHD | 1.528 | 0.337 | 2.719 | 0.013 |
| CD16 | S2_UT | AF | 1.559 | 0.336 | 2.782 | 0.013 |
| CD16 | S2_UT | HFpEF | 1.534 | 0.324 | 2.743 | 0.014 |
| CD16 | S2_UT | DLP | 1.528 | 0.309 | 2.748 | 0.015 |
| CD16 | S2_UT | Age | 1.463 | 0.234 | 2.691 | 0.020 |
| CD16 | S2_UT | BMI | 1.445 | 0.227 | 2.663 | 0.021 |
| CD16 | S2_UT | DM | 1.431 | 0.182 | 2.680 | 0.025 |
| COL1A2 | S2_UT | Age | 0.813 | 0.180 | 1.446 | 0.012 |
| COL1A2 | S2_UT | HTN | 0.783 | 0.158 | 1.408 | 0.015 |
| COL1A2 | S2_UT | VHD | 0.779 | 0.157 | 1.401 | 0.015 |
| COL1A2 | S2_UT | HFpEF | 0.782 | 0.157 | 1.407 | 0.015 |
| COL1A2 | S2_UT | CAD | 0.762 | 0.144 | 1.379 | 0.016 |
| COL1A2 | S2_UT | DLP | 0.760 | 0.137 | 1.383 | 0.017 |
| COL1A2 | S2_UT | BMI | 0.748 | 0.122 | 1.373 | 0.020 |
| COL1A2 | S2_UT | AF | 0.726 | 0.104 | 1.348 | 0.023 |
| COL1A2 | S2_UT | DM | 0.661 | 0.024 | 1.299 | 0.042 |
| DEFA3 | S2_UT | CAD | 2.396 | 0.479 | 4.313 | 0.015 |
| DEFA3 | S2_UT | VHD | 2.356 | 0.448 | 4.264 | 0.016 |
| DEFA3 | S2_UT | HFpEF | 2.332 | 0.359 | 4.304 | 0.021 |
| DEFA3 | S2_UT | DLP | 2.313 | 0.325 | 4.301 | 0.023 |
| DEFA3 | S2_UT | HTN | 2.266 | 0.275 | 4.256 | 0.026 |
| DEFA3 | S2_UT | BMI | 2.176 | 0.175 | 4.177 | 0.033 |
| DEFA3 | S2_UT | Age | 2.124 | 0.109 | 4.139 | 0.039 |
| DEFA3 | S2_UT | DM | 2.144 | 0.087 | 4.201 | 0.041 |
| DEFA3 | S2_UT | AF | 2.064 | 0.075 | 4.053 | 0.042 |
| CD14 | S3_UT | HFpEF | 0.983 | 0.249 | 1.716 | 0.009 |
| CD14 | S3_UT | CAD | 0.916 | 0.219 | 1.614 | 0.011 |
| CD14 | S3_UT | HTN | 0.939 | 0.212 | 1.666 | 0.012 |
| CD14 | S3_UT | AF | 0.915 | 0.176 | 1.654 | 0.016 |
| CD14 | S3_UT | VHD | 0.890 | 0.169 | 1.612 | 0.016 |
| CD14 | S3_UT | DLP | 0.900 | 0.163 | 1.637 | 0.017 |
| CD14 | S3_UT | BMI | 0.885 | 0.149 | 1.621 | 0.019 |
| CD14 | S3_UT | Age | 0.882 | 0.135 | 1.629 | 0.021 |
| CD14 | S3_UT | DM | 0.843 | 0.099 | 1.588 | 0.027 |
| CD16 | S3_UT | HFpEF | 1.647 | 0.372 | 2.922 | 0.012 |
| CD16 | S3_UT | HTN | 1.594 | 0.338 | 2.850 | 0.013 |
| CD16 | S3_UT | CAD | 1.529 | 0.312 | 2.747 | 0.014 |
| CD16 | S3_UT | AF | 1.576 | 0.304 | 2.847 | 0.016 |
| CD16 | S3_UT | DLP | 1.546 | 0.276 | 2.815 | 0.018 |
| CD16 | S3_UT | VHD | 1.494 | 0.265 | 2.722 | 0.018 |
| CD16 | S3_UT | BMI | 1.522 | 0.251 | 2.794 | 0.019 |
| CD16 | S3_UT | Age | 1.471 | 0.190 | 2.752 | 0.025 |
| CD16 | S3_UT | DM | 1.457 | 0.180 | 2.734 | 0.026 |
| COL1A2 | S3_UT | HFpEF | 0.641 | 0.007 | 1.274 | 0.047 |
| COL1A2 | S3_UT | VHD | 0.627 | 0.000 | 1.254 | 0.050 |
| COL1A2 | S3_UT | HTN | 0.626 | -0.002 | 1.255 | 0.051 |
| COL1A2 | S3_UT | CAD | 0.623 | -0.002 | 1.247 | 0.051 |
| COL1A2 | S3_UT | BMI | 0.623 | -0.004 | 1.250 | 0.051 |
| COL1A2 | S3_UT | DLP | 0.619 | -0.007 | 1.245 | 0.053 |
| COL1A2 | S3_UT | Age | 0.620 | -0.016 | 1.255 | 0.056 |
| COL1A2 | S3_UT | AF | 0.595 | -0.031 | 1.220 | 0.062 |
| COL1A2 | S3_UT | DM | 0.555 | -0.074 | 1.185 | 0.083 |
| DEFA3 | S3_UT | HFpEF | 2.158 | 0.102 | 4.215 | 0.040 |
| DEFA3 | S3_UT | CAD | 1.963 | -0.007 | 3.932 | 0.051 |
| DEFA3 | S3_UT | VHD | 1.896 | -0.038 | 3.831 | 0.055 |
| DEFA3 | S3_UT | DLP | 1.969 | -0.082 | 4.020 | 0.060 |
| DEFA3 | S3_UT | HTN | 1.962 | -0.100 | 4.023 | 0.062 |
| DEFA3 | S3_UT | BMI | 1.928 | -0.135 | 3.990 | 0.067 |
| DEFA3 | S3_UT | AF | 1.818 | -0.241 | 3.877 | 0.083 |
| DEFA3 | S3_UT | DM | 1.823 | -0.264 | 3.910 | 0.086 |
| DEFA3 | S3_UT | Age | 1.789 | -0.291 | 3.868 | 0.091 |
| **B: Male population** | | | | | | |
| **Marker** | **ECG** | **clinical factor** | **beta** | **ci_low** | **ci_high** | **p_value** |
| CD14 | S2_UT | HTN | 0.640 | 0.046 | 1.234 | 0.035 |
| CD14 | S2_UT | CAD | 0.568 | 0.007 | 1.129 | 0.047 |
| CD14 | S2_UT | AF | 0.575 | -0.019 | 1.169 | 0.058 |
| CD14 | S2_UT | BMI | 0.569 | -0.020 | 1.159 | 0.058 |
| CD14 | S2_UT | Age | 0.564 | -0.029 | 1.156 | 0.062 |
| CD14 | S2_UT | DLP | 0.567 | -0.033 | 1.168 | 0.064 |
| CD14 | S2_UT | HFpEF | 0.551 | -0.037 | 1.138 | 0.066 |
| CD14 | S2_UT | DM | 0.550 | -0.043 | 1.142 | 0.069 |
| CD14 | S2_UT | VHD | 0.516 | -0.070 | 1.102 | 0.084 |
| CD16 | S2_UT | HTN | 1.208 | 0.172 | 2.245 | 0.023 |
| CD16 | S2_UT | AF | 1.141 | 0.104 | 2.178 | 0.032 |
| CD16 | S2_UT | CAD | 1.098 | 0.098 | 2.098 | 0.032 |
| CD16 | S2_UT | DLP | 1.147 | 0.094 | 2.201 | 0.033 |
| CD16 | S2_UT | BMI | 1.114 | 0.083 | 2.146 | 0.035 |
| CD16 | S2_UT | Age | 1.107 | 0.073 | 2.140 | 0.036 |
| CD16 | S2_UT | DM | 1.095 | 0.061 | 2.129 | 0.038 |
| CD16 | S2_UT | HFpEF | 1.079 | 0.047 | 2.110 | 0.041 |
| CD16 | S2_UT | VHD | 0.988 | -0.037 | 2.013 | 0.059 |
| COL1A2 | S2_UT | VHD | 0.552 | 0.038 | 1.066 | 0.036 |
| COL1A2 | S2_UT | BMI | 0.538 | 0.028 | 1.048 | 0.039 |
| COL1A2 | S2_UT | CAD | 0.531 | 0.024 | 1.039 | 0.040 |
| COL1A2 | S2_UT | HFpEF | 0.536 | 0.023 | 1.049 | 0.041 |
| COL1A2 | S2_UT | Age | 0.534 | 0.021 | 1.047 | 0.041 |
| COL1A2 | S2_UT | DM | 0.507 | 0.000 | 1.014 | 0.050 |
| COL1A2 | S2_UT | HTN | 0.520 | -0.001 | 1.040 | 0.050 |
| COL1A2 | S2_UT | AF | 0.501 | -0.007 | 1.010 | 0.053 |
| COL1A2 | S2_UT | DLP | 0.492 | -0.025 | 1.009 | 0.062 |
| DEFA3 | S2_UT | CAD | 1.741 | 0.161 | 3.321 | 0.031 |
| DEFA3 | S2_UT | DLP | 1.820 | 0.166 | 3.474 | 0.031 |
| DEFA3 | S2_UT | HTN | 1.779 | 0.113 | 3.445 | 0.037 |
| DEFA3 | S2_UT | BMI | 1.720 | 0.081 | 3.358 | 0.040 |
| DEFA3 | S2_UT | Age | 1.705 | 0.068 | 3.342 | 0.041 |
| DEFA3 | S2_UT | DM | 1.695 | 0.052 | 3.338 | 0.043 |
| DEFA3 | S2_UT | HFpEF | 1.671 | 0.046 | 3.297 | 0.044 |
| DEFA3 | S2_UT | AF | 1.635 | 0.004 | 3.267 | 0.049 |
| DEFA3 | S2_UT | VHD | 1.511 | -0.069 | 3.092 | 0.061 |
| CD14 | S3_UT | HTN | 0.584 | -0.042 | 1.209 | 0.067 |
| CD14 | S3_UT | HFpEF | 0.519 | -0.099 | 1.136 | 0.099 |
| CD14 | S3_UT | BMI | 0.508 | -0.120 | 1.136 | 0.111 |
| CD14 | S3_UT | DM | 0.502 | -0.122 | 1.126 | 0.114 |
| CD14 | S3_UT | DLP | 0.496 | -0.131 | 1.123 | 0.119 |
| CD14 | S3_UT | Age | 0.490 | -0.136 | 1.115 | 0.124 |
| CD14 | S3_UT | AF | 0.488 | -0.136 | 1.113 | 0.124 |
| CD14 | S3_UT | CAD | 0.457 | -0.135 | 1.049 | 0.129 |
| CD14 | S3_UT | VHD | 0.411 | -0.207 | 1.028 | 0.190 |
| CD16 | S3_UT | HTN | 1.053 | -0.056 | 2.163 | 0.062 |
| CD16 | S3_UT | HFpEF | 0.943 | -0.157 | 2.044 | 0.092 |
| CD16 | S3_UT | DM | 0.947 | -0.163 | 2.057 | 0.093 |
| CD16 | S3_UT | DLP | 0.935 | -0.175 | 2.046 | 0.098 |
| CD16 | S3_UT | Age | 0.918 | -0.189 | 2.025 | 0.103 |
| CD16 | S3_UT | AF | 0.907 | -0.196 | 2.011 | 0.106 |
| CD16 | S3_UT | BMI | 0.905 | -0.210 | 2.021 | 0.110 |
| CD16 | S3_UT | CAD | 0.831 | -0.232 | 1.894 | 0.124 |
| CD16 | S3_UT | VHD | 0.704 | -0.380 | 1.787 | 0.200 |
| COL1A2 | S3_UT | DM | 0.397 | -0.130 | 0.925 | 0.138 |
| COL1A2 | S3_UT | CAD | 0.384 | -0.147 | 0.915 | 0.154 |
| COL1A2 | S3_UT | BMI | 0.387 | -0.148 | 0.922 | 0.155 |
| COL1A2 | S3_UT | HFpEF | 0.382 | -0.151 | 0.915 | 0.158 |
| COL1A2 | S3_UT | AF | 0.377 | -0.151 | 0.906 | 0.160 |
| COL1A2 | S3_UT | Age | 0.378 | -0.154 | 0.911 | 0.161 |
| COL1A2 | S3_UT | HTN | 0.375 | -0.166 | 0.916 | 0.172 |
| COL1A2 | S3_UT | VHD | 0.370 | -0.166 | 0.907 | 0.174 |
| COL1A2 | S3_UT | DLP | 0.366 | -0.167 | 0.898 | 0.176 |
| DEFA3 | S3_UT | HFpEF | 1.157 | -0.572 | 2.886 | 0.187 |
| DEFA3 | S3_UT | HTN | 1.179 | -0.597 | 2.955 | 0.190 |
| DEFA3 | S3_UT | DLP | 1.144 | -0.600 | 2.887 | 0.196 |
| DEFA3 | S3_UT | DM | 1.108 | -0.641 | 2.856 | 0.212 |
| DEFA3 | S3_UT | Age | 1.095 | -0.649 | 2.838 | 0.215 |
| DEFA3 | S3_UT | AF | 1.084 | -0.656 | 2.823 | 0.219 |
| DEFA3 | S3_UT | CAD | 1.013 | -0.660 | 2.685 | 0.232 |
| DEFA3 | S3_UT | BMI | 1.065 | -0.694 | 2.824 | 0.232 |
| DEFA3 | S3_UT | VHD | 0.721 | -0.934 | 2.376 | 0.389 |

AF: atrial fibrillation; BMI: body mass index; CAD: coronary artery disease; ci: 95% confidence interval; COL1A2: collagen type I alpha 2 chain; DEFA3: defensin alpha 3; DLP: Dyslipidaemia; DM: diabetes mellitus; HFpEF: heart failure preserved ejection fraction; HTN: hypertension; S2 UT: S-wave upstroke time in V2; S3 UT: S-wave upstroke time in V3; VHD: valvular

## 2.2 Supplementary Figures

**Supplementary Figure 1.** Scree, Contributions, and Cos² plots for PCA results in the depolarization group. A: scree plot showing the percentage of explained variance for each principal component. Bars represent the percentage of variance explained by each component, and the line connects eigenvalues across dimensions. B-E: contribution of variables to PC1-4. Bars represent the percentage contribution of each variable to the corresponding principal component. F: Cos² values of variables across the first four principal components. Bars represent the squared cosine (cos²) for each variable.

QRSd: QRS dispersion; QRS R/L: QRS right (V1+V2+V3)-to-left (V4+V5+V6) ratio; R1 A: R-wave amplitude in V1; R2 A: R-wave amplitude in V2; R3 A: R-wave amplitude in V3; R1 PT: R-wave peak time in V1; R2 PT: R-wave peak time in V2; R3 PT: R-wave peak time in V3; R/S 1: R/S amplitude ratio in V1; R/S 2: R/S amplitude ratio in V2; R/S 3: R/S amplitude ratio in V3; S1 A: S-wave amplitude in V1; S2 A: S-wave amplitude in V2; S3 A: S-wave amplitude in V3; S1 UT: S-wave upstroke time in V1; S2 UT: S-wave upstroke time in V2; S3 UT: S-wave upstroke time in V3; WQRS/NQRS: the widest QRS (in V1 or V2)/the narrowest QRS (in V5 or V6).

**Supplementary Figure 2.** Scree, Contributions, and Cos² plots for PCA results in the repolarization group. A: scree plot showing the percentage of explained variance for each principal component. Bars represent the percentage of variance explained by each component, and the line connects eigenvalues across dimensions. B-D: contribution of variables to PC1-3. Bars represent the percentage contribution of each variable to the corresponding principal component. E: Cos² values of variables across the first four principal components. Bars represent the squared cosine (cos²) for each variable.

dTpe: dispersion of T peak - T end; JTc: corrected JT; JTcd: dispersion of corrected JT; JT R/L: JT right (V1+V2+V3)-to-left (V4+V5+V6) ratio; JTc R/L: corrected JT right (V1+V2+V3)-to-left (V4+V5+V6) ratio; QTc: corrected QT; QTcd: dispersion of corrected QT; QT R/L: QT right (V1+V2+V3)-to-left (V4+V5+V6) ratio; QTc R/L: corrected QT right (V1+V2+V3)-to-left (V4+V5+V6) ratio; Tpe: T peak - T end; Tpe/QT R/L: Tpe/QT right (V1+V2+V3)-to-left (V4+V5+V6) ratio; Tpe/QTc R/L: Tpe/QTc right (V1+V2+V3)-to-left (V4+V5+V6) ratio; Tpe R/L: Tpe right (V1+V2+V3)-to-left (V4+V5+V6) ratio.

**Supplementary Figure 3.** Variables and individuals’ distribution in PCA space in the depolarization group. A: PCA variable projection on the first two components. Variables that point in similar directions share related electrophysiological characteristics, while the length of each vector reflects the strength of its contribution. The colour scale indicates the quality of representation (cos²), with higher values showing variables that are well captured by the PC1-PC2 space. B: PCA loading plot combining variable loadings and individual factor scores on the first two components. Arrows represent variable loadings, while points represent individual samples.

QRSd: QRS dispersion; QRS R/L: QRS right (V1+V2+V3)-to-left (V4+V5+V6) ratio; R1 A: R-wave amplitude in V1; R2 A: R-wave amplitude in V2; R3 A: R-wave amplitude in V3; R1 PT: R-wave peak time in V1; R2 PT: R-wave peak time in V2; R3 PT: R-wave peak time in V3; R/S 1: R/S amplitude ratio in V1; R/S 2: R/S amplitude ratio in V2; R/S 3: R/S amplitude ratio in V3; S1 A: S-wave amplitude in V1; S2 A: S-wave amplitude in V2; S3 A: S-wave amplitude in V3; S1 UT: S-wave upstroke time in V1; S2 UT: S-wave upstroke time in V2; S3 UT: S-wave upstroke time in V3; WQRS/NQRS: the widest QRS (in V1 or V2)/the narrowest QRS (in V5 or V6).

**Supplementary Figure 4.** Variables and individuals’ distribution in PCA space in the repolarization group. A: PCA variable projection on the first two components. Variables that point in similar directions share related electrophysiological characteristics, while the length of each vector reflects the strength of its contribution. The colour scale indicates the quality of representation (cos²), with higher values showing variables that are well captured by the PC1-PC2 space. B: PCA loading plot combining variable loadings and individual factor scores on the first two components. Arrows represent variable loadings, while points represent individual samples.

dTpe: dispersion of T peak - T end; JTc: corrected JT; JTcd: dispersion of corrected JT; JT R/L: JT right (V1+V2+V3)-to-left (V4+V5+V6) ratio; JTc R/L: corrected JT right (V1+V2+V3)-to-left (V4+V5+V6) ratio; QTc: corrected QT; QTcd: dispersion of corrected QT; QT R/L: QT right (V1+V2+V3)-to-left (V4+V5+V6) ratio; QTc R/L: corrected QT right (V1+V2+V3)-to-left (V4+V5+V6) ratio; Tpe: T peak - T end; Tpe/QT R/L: Tpe/QT right (V1+V2+V3)-to-left (V4+V5+V6) ratio; Tpe/QTc R/L: Tpe/QTc right (V1+V2+V3)-to-left (V4+V5+V6) ratio; Tpe R/L: Tpe right (V1+V2+V3)-to-left (V4+V5+V6) ratio.
